# Clinical significance of YAP1 activation in head and neck squamous cell carcinoma

**DOI:** 10.18632/oncotarget.22666

**Published:** 2017-11-27

**Authors:** Young-Gyu Eun, Dongjin Lee, Young Chan Lee, Bo Hwa Sohn, Eui Hyun Kim, Sun Young Yim, Kee Hwan Kwon, Ju-Seog Lee

**Affiliations:** ^1^ Department of Systems Biology, The University of Texas MD Anderson Cancer Center, Houston, Texas, USA; ^2^ Department of Otolaryngology-Head and Neck Surgery, School of Medicine, Kyung Hee University, Seoul, Republic of Korea; ^3^ Department of Otolaryngology-Head and Neck Surgery, School of Medicine, Hallym University, Seoul, Republic of Korea; ^4^ Department of Neurosurgery, Severance Hospital, Brain Tumor Center, Yonsei University College of Medicine, Seoul, Republic of Korea; ^5^ Division of Gastroenterology and Hepatology, Department of Internal Medicine, Korea University College of Medicine, Seoul, Republic of Korea

**Keywords:** YAP1, gene signature, head and neck cancer, prognosis

## Abstract

By analyzing the genomic data of head and neck squamous cell cancer (HNSCC), we investigated clinical significance of YAP1 activation. Copy number and mRNA expression of YAP1 were analyzed together to assess clinical relevance of YAP1 activation in HNSCC. The clinical significance of YAP1 activation was further validated in four independent test cohorts. We also assessed the correlation of YAP1 activation with genomic alterations such as copy number alteration, somatic mutation, and miRNA expression. The YAP1-activated (YA) subgroup showed worse prognosis for HNSCC as tested and validated in five cohorts. In a multivariate risk analysis, the YAP1 signature was the most significant predictor of overall survival. The YAP1-inactivated (YI) subgroup was associated with HPV-positive status. In multiplatform analysis, YA tumors had gain of EGFR and SNAI2; loss of tumor-suppressor genes such as CSMD1, CDKN2A, NOTCH1, and SMAD4; and high mutation rates of TP53 and CDKN2A. YI tumors were characterized by gain of PIK3CA, SOX2, and TP63; deletion of 11q23.1; and high mutation rates of NFE2L2, PTEN, SYNE1, and NSD1. YA tumors also showed weaker immune activity as reflected in low IFNG composite scores and YAP1 activity is negatively associated with potential response to treatment of pembrolizumab. In conclusion, activation of YAP1 is associated with worse prognosis of patients with HNSCC and potential resistance to immunotherapy.

## INTRODUCTION

Head and neck squamous cell carcinoma (HNSCC) is the sixth leading type of cancer worldwide, with an annual incidence of approximately 600,000 cases and a mortality rate of 40% to 50% [1, 2]. The major known risk factors are environmental exposures to tobacco products, alcohol, and infection with high-risk human papillomaviruses (HPV) [3]. Despite advances in our knowledge of the epidemiology and pathogenesis of HNSCC and the use of radiation treatment and functional surgery for this disease, survival rates have not improved over the past 40 years [4].

The Hippo pathway is a major tumor-suppressor pathway in many cancers and, in general, has important regulatory functions in cell proliferation, cell survival, cell competition, and maintenance of a stem cell phenotype [5]. The Hippo pathway consists of a regulatory serine-threonine kinase module and a transcriptional module. The kinase module includes mammalian STE20-like protein kinase 1 (MST1) and MST2, large tumor suppressor 1 (LATS1) and LATS2, together with the adaptor proteins Salvador homologue 1 (SAV1), MOB kinase activator 1A (MOB1A), and MOB1B [6-11]. These inhibitory kinase modules regulate tissue growth by suppressing the transcription module such as the Yes-associated protein-1 (YAP1) and transcriptional co-activator with PDZ-binding motif (TAZ) [12]. YAP1 is a key conduit for Hippo pathway regulation and output [5]. Hyperactivation of YAP1 is widespread in cancers. Previous studies showed that cancer features such as cancer stem cell properties, epithelial-mesenchymal transition (EMT), increased migration, and metastasis are regulated by YAP1 [11]. In a study of the mutational landscape across 12 major cancer types, significantly mutated genes of hippo signaling were found in several cancers included HNSCC [13].

In oral squamous cell carcinoma, nuclear YAP1 accumulation marked premalignant dysplastic regions of the oral epithelium and YAP1 promoted tumorigenic phenotypes and a transcriptional program associated with tumor progression [14]. YAP1 expression in combination with p63 can facilitate identification of HNSCC tumors from hyperplastic and benign tissues [15]. Furthermore, YAP1 upregulation is known to be related with resistance to anticancer drugs such as docetaxel, cisplatin, and cetuximab in several cancers [16-18]. In oral squamous cell carcinoma cell lines, nuclear translocation of YAP1 correlated with the acquisition of cisplatin resistance [19]. In HNSCC, amplification of the YAP1 gene was linked to cetuximab resistance in cell lines [20]. However, the clinical relevance of YAP1 activation has still not been examined in HNSCC.

In the current study, we systematically characterized the genomic data from multiple cohorts of patients with HNSCC and found a molecular subtype characterized by YAP1 activation and poor prognosis. We analyzed the gene alterations associated with YAP1 using multiplatforms. We further showed that YAP1 may play roles in resistant to immunotherapy.

## RESULTS

### Activation of YAP1 in HNSCC

**Figure 1:**
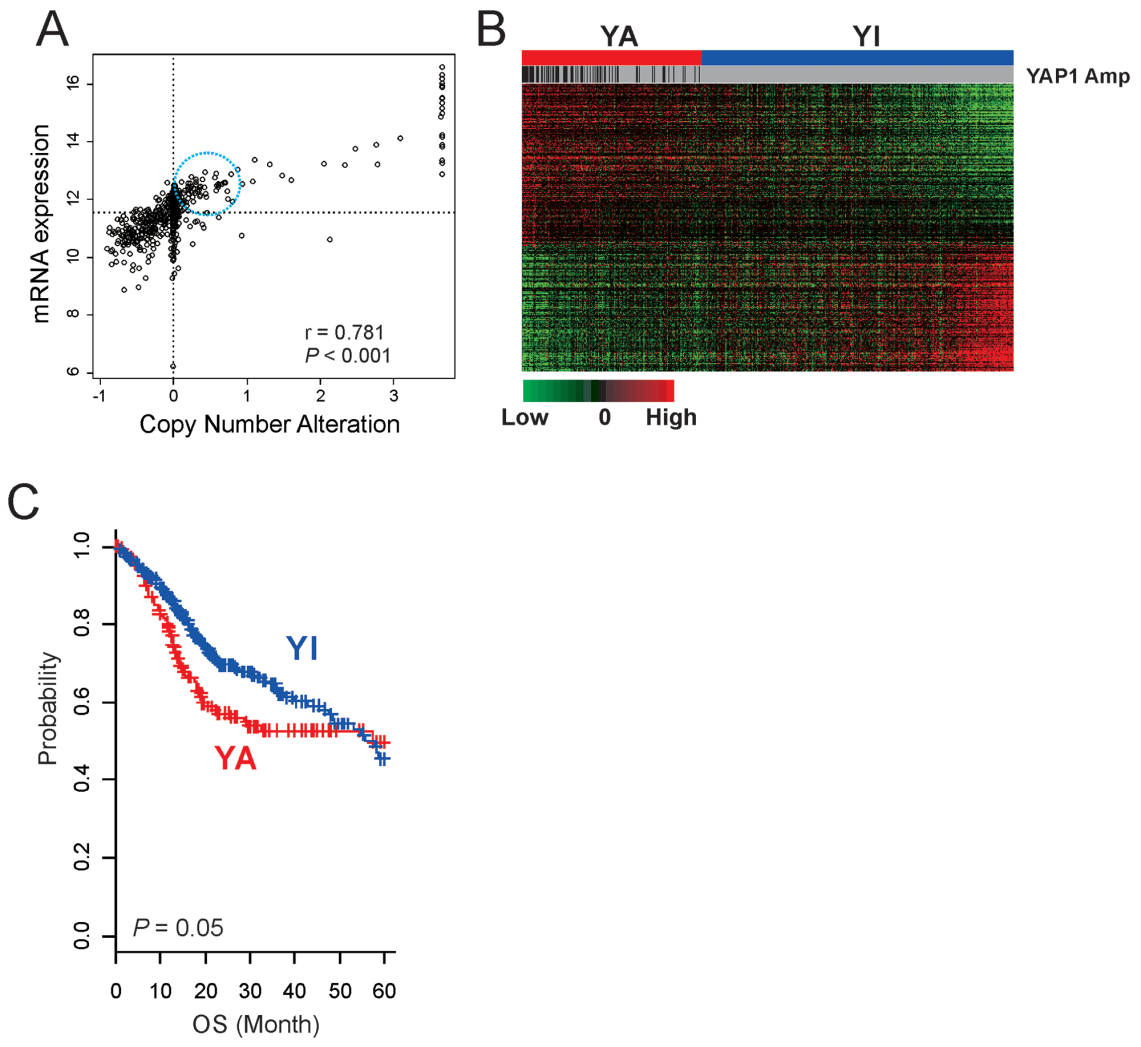
Activation of YAP1 in HNSCC **(A)** Scatter plots between mRNA expression and copy number alteration of YAP1 in TCGA cohort. The expression of YAP1 was substantially higher in many tumors without YAP1 amplification (in circle). **(B)** Expression patterns of 292 genes in the YAP1 signature. The data are presented in matrix format, in which each row represents an individual gene and each column represents a tissue specimen. Each cell in the matrix represents the level of expression of a gene feature in an individual sample. The red and green cells reflect relatively high and low expression levels, respectively. YA, YAP1 activated; YI, YAP1 inactivated. **(C)** Kaplan-Meier plots of the YA and YI patients in TCGA cohort. Overall survival time of patients with the YA subtype was significantly worse than that of patients with the YI subtype. P values were calculated using log-rank tests. +, censored data.

Although activation of YAP1 has been reported in many cancers [5, 16, 18, 21-24], its relative activity across all cancer types has not been systematically examined. Because amplification is one common activation mechanism of YAP1 [25, 26], we first examined copy number alteration of YAP1 in 26 cancers by using genome copy number data from TCGA. HNSCC is the second most common YAP1-amplified cancer type (8.6%) after cervical cancer (12.6%) (Supplementary Figure 1A), suggesting that YAP1 activation might be a critical genetic event for development of HNSCC. Expression of YAP1 was significantly correlated with copy number alteration (Figure 1A, r = 0.781, P < 0.001), further supporting functional roles of YAP1 in the development of HNSCC. Interestingly, expression of YAP1 was substantially higher in many tumors without YAP1 amplification, suggesting that copy number alteration is not the only mechanism of YAP1 activation in HNSCC (Figure 1A).

Because the best-known molecular activity of YAP1 is transcription activation, we sought to identify potential downstream targets of YAP1 by finding genes with expression significantly correlated with copy number alterations (P < 0.001 and Pearson correlation coefficient > 0.2 or < –0.2), yielding 652 genes (copy number-associated genes). We next constructed a prediction model with these potential downstream target genes to estimate YAP1 activity in HNSCC by using the BCCP model. Briefly, tumors were first divided into two groups according to copy number alterations: YAP1-high (GISTIC score >2) and YAP1-low (GISTIC score <2). As expectedly, the vast majority of YAP1-high tumors showed a high probability (>0.5) of YAP1 activity when BCCP was applied to gene expression data. Interestingly, many tumors without YAP1 amplification also showed a high probability of YAP1 activity (Figure 1A), further supporting our notion that YAP1 amplification may not be the only mechanism for YAP1 activation in HNSCC. Because many tumors showed high YAP1 expression without YAP1 amplification, we also identified genes with expression significantly correlated with mRNA expression of YAP1 (P < 0.001 and Pearson correlation coefficient > 0.2 or < –0.2). This search yielded 4552 genes (mRNA-associated genes). To identify key downstream targets of YAP1 in HNSCC, we further selected 292 genes that were shared in both gene lists (Supplementary Figure 1B, Supplementary Table 1). To assess the clinical relevance of YAP1 activation in HNSCC, patients were re-stratified according to BCCP probability: YAP1-active (YA >0.5) and YAP1-inactive (YI <0.5) (Figure 1B). Overall survival (OS) time of patients with the YA subtype was significantly worse than that of patients with the YI subtype, suggesting that YAP1 activation is significantly associated with poor prognosis in HNSCC (Figure 1C).

### YAP1 signature was associated with the prognosis of HNSCC

**Figure 2:**
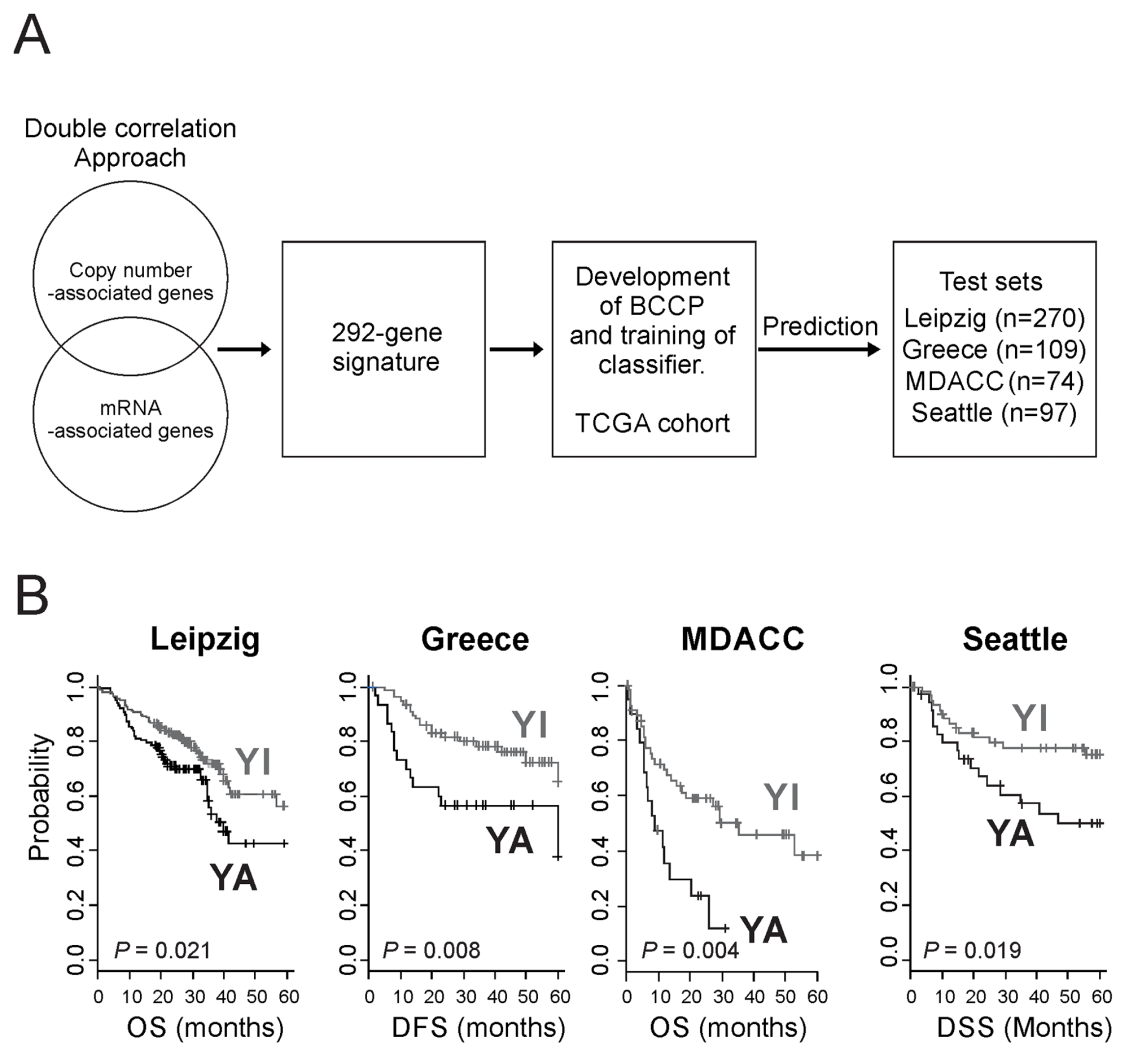
Construction of a prediction model using YAP1 gene signature and clinical significance of YAP1 activation in HNSCC **(A)** Schematic diagram of the strategy used to construct the prediction model and evaluate predicted HNSCC outcomes according to the gene expression signature. **(B)** Kaplan-Meier plots of the YA and YI patients in four independent test cohorts. P values were calculated using log-rank tests. +, censored data.

Having shown that gene expression signature accurately reflecting activation of YAP1 and had a significant association with prognosis, we next sought to validate its association in the independent cohorts (Leipzig, Greek, MD Anderson, and Seattle cohorts) (Figure 2A). Patients in these cohorts were stratified to the YA or YI group by BCCP classifier (Supplementary Figure 2, Table 1). When this stratification was applied to the Leipzig cohort, 115 (42.6%) of 270 patients were classified as YA (Table 1), and their prognosis was significantly worse than for patients in the YI group (OS 27.7% vs. 38.3% at 5 years, P = 0.021) (Figure 2B). Of 109 patients in the Greek cohort, 30 (27.5%) were classified as YA, and the patients with the YA subtype had a significantly worse disease-free survival than did patients with the YI subtype (37.8% vs. 65.1% at 5 years, P = 0.008) (Figure 2B). In the MD Anderson cohort, 19 (25.7%) of 74 patients were classified as YA and had significantly worse disease-free survival than those with the YI subtype (43.3% vs. 75.8%, P = 0.008) (Figure 2B). Consistent with previous observation, patients classified as YA subtype in the Seattle cohort (35 patients or 36.1%) also showed significantly poorer prognosis (P = 0.019) (Figure 2B).

**Table 1:** Patient’s characteristics in 5 cohort

	TCGA cohort	Leipzig cohort	Greece cohort	MDACC cohort	Seattle cohort
Number of patients	513	270	109	74	97
Gender					
Male	370 (73.7%)	223 (82.6%)	104 (95.4%)	58 (78.4%)	66 (68.0%)
Female	132 (26.3%)	47 (17.4%)	5 (4.6%)	16 (21.6%)	31 (32.0%)
Age (mean ± SD)	60.9 ± 11.9	60.1 ± 10.0	63.3 ± 10.1	58.1 ± 13.6	NA
Anatomic site					
Oral cavity	301 (60.0%)	83 (30.7%)	0	71 (95.9%)	86 (88.7%)
Oropharynx	79 (15.7%)	102 (37.8%)	0	3 (4.1%)	11 (11.3%)
Larynx	113 (22.5%)	48 (17.8%)	109 (100%)	0	0
Hypopharynx	9 (1.8%)	33 (12.2%)	0	0	0
others	0	4 (1.5%)	0	0	0
Primary tumor					
T1	33 (6.8%)	35 (13.0%)	NA	3 (4.1%)	NA
T2	147 (30.2%)	80 (29.6%)	NA	27 (36.5%)	NA
T3	129 (26.5%)	58 (21.5%)	NA	28 (37.8%)	NA
T4	178 (36.6%)	97 (35.9%)	NA	16 (21.6%)	NA
Regional lymph node					
N0	238 (49.5%)	94 (34.8%)	42 (56.8%)	NA	NA
N1	79 (16.4%)	32 (11.9%)	13 (17.6%)	NA	NA
N2	155 (32.2%)	132 (48.9%)	19 (25.7%)	NA	NA
N3	9 (1.9%)	12 (4.4%)	0	NA	NA
Stage					
I	20 (4.1%)	18 (6.7%)	12 (11.0%)	3 (4.1%)	30 (30.9%)
II	96 (19.6%)	37 (13.7%)	18 (16.5%)	16 (21.6%)	11 (11.3%)
III	101 (20.7%)	37 (13.7%)	36 (33.0%)	15 (20.3%)	15 (15.5%)
IV	272 (55.6%)	178 (65.9%)	43 (39.4%)	40 (54.1%)	41 (42.3%)
HPV status					
Positive	68 (19.9%)	60 (23.4%)	NA	NA	0
Negative	274 (80.1%)	196 (76.6%)	NA	NA	97 (100%)
Tobacco use					
Never	114 (23.3%)	48 (17.8%)	1 (0.9%)	15 (20.3%)	NA
Yes	376 (76.7%)	222 (82.2%)	108 (99.1%)	59 (79.7%)	NA
Alcohol use					
Never	154 (42.1%)	31 (11.5%)	51 (46.8%)	NA	NA
Yes	212 (57.9%)	239 (88.5%)	58 (53.2%)	NA	NA
YAP1 signature					
YAHSC	187 (36.5%)	115 (42.6%)	30 (27.5%)	19 (25.7%)	35 (36.1%)
YIHSC	326 (63.5%)	155 (57.4%)	79 (72.5%)	55 (74.3%)	62 (63.9%)

We next performed analyses to determine whether the prognostic effect of the YAP1 signature was independent of other clinical variables. Three cohorts (TCGA, Leipzig, and MD Anderson) with available OS data were pooled for a Cox proportional hazards model (n = 808). In univariate analysis, YAP1 signature (YA group vs. YI group), sex, age (<60 years old vs. ≥60 years old), anatomic site (oropharynx vs. non-oropharynx), and primary tumor (T4 vs. non-T4) were significant prognostic factors (Table 2). To address a potential confounding effect, we performed a multivariate risk analysis using the Cox proportional hazards model and found that the YAP1 signature was the most significant predictor of OS (hazard ratio 1.31, 95% confidence interval 1.03-1.67, P = 0.028).

**Table 2:** Univariate and multivariate Cox Proportinal Hazard Regression Analyses of variables affecting 5-year overall survival rate (patients data: TCGA cohort, Leipzig cohort, MDACC cohort; n=808)

	Univariate		Multivariate	
	HR (95% CI)	P-value	HR (95% CI)	P-value
YAP1 signature (YAP1 activated)	1.41 (1.12-1.77)	0.004	1.31 (1.03-1.67)	0.028
Gender (male)	0.77 (0.60-1.0)	0.05	0.85 (0.65-1.12)	0.247
Age (≥ 60 y)	1.30 (1.04-1.64)	0.024	1.26 (0.99-1.61)	0.066
Anatomic site (oropharynx)	0.54 (0.32-0.92)	0.025	0.63 (0.37-1.10)	0.104
Primary tumor (T4)	2.03 (1.00-4.12)	0.049	1.81 (0.88-3.73)	0.108
Regional lymph node (N+)	1.02 (0.81-1.28)	0.9	1.01 (0.76-1.34)	0.971
Stage (stage III & IV)	1.21 (0.91-1.62)	0.19	1.08 (0.74-1.56)	0.695

Next, we assessed whether YAP1 activation may have a potential association with the patient’s response to radiation therapy. We pooled the patient data from TCGA and MD Anderson cohorts with available radiation data (n = 458). For the patients who underwent radiation therapy, the survival of patients with the YA subtype was significantly worse than for patients with the YI subtype (P = 0.03) (Supplementary Figure 3A). For the YA group, radiation therapy did not improve the survival rate (P = 0.15) (Supplementary Figure 3B). However, radiation therapy improved the survival rate of the YI group (P = 0.04) (Supplementary Figure 3C), suggesting that YAP1 activation might be associated with radioresistance in HNSCC.

### Copy number alteration associated with YAP1 activation

**Figure 3:**
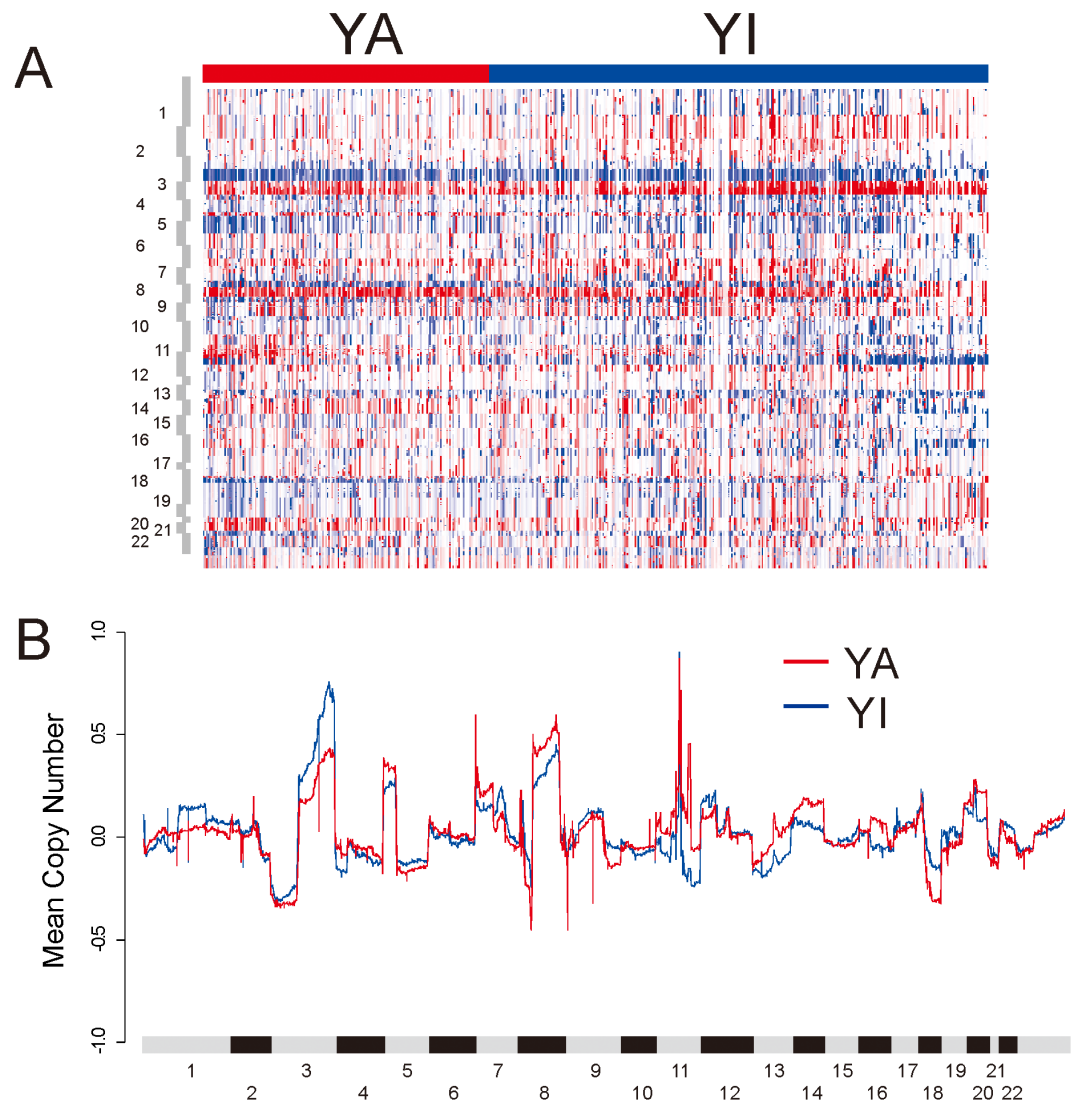
DNA copy number alterations in TCGA HNSCC cohort **(A)** Heat map of copy number alterations of tumors stratified by YAP1 subtype. **(B)** Copy number gains and losses in YA and YI subtypes. For each gene, the mean of the segmented GISTIC copy number values, in YA and YI subtypes were computed and plotted in genomic order.

To further examine the genomic properties associated with YAP1 activation, we compared the copy number alteration between the YA and YI groups (Figure 3). Somatic copy numbers were obtained for 513 samples in TCGA cohort. When frequency of significantly reoccurring alterations was compared using Fisher’s exact test, the YA group had amplification of 7p11.2 (EGFR) and 8q11.21 (SNAI2). The YA group had deletion of 8p23 (CSMD1), 9p21.3 (CDKN2A), 9q34.3 (NOTCH1), and 18q21.2 (SMAD4). CSMD1, CDKN2A, NOTCH1, and SMAD4 are known to be tumor-suppressor genes. The YI group was distinguished by amplification of 3q26/28 (PIK3CA, SOX2, and TP63) and deletion of 11q23.1 (Supplementary Table 2). Gains in the 3q, 5p, and 8q chromosomal regions were features of HNSCC and lung squamous cell carcinoma [27]. Interestingly, while gain in the 3q region was higher in the YI group, gain in the 8q region was higher for the YA group. There was no difference in 5p between the two groups.

### Somatic mutation associated with YAP1 activation

**Figure 4:**
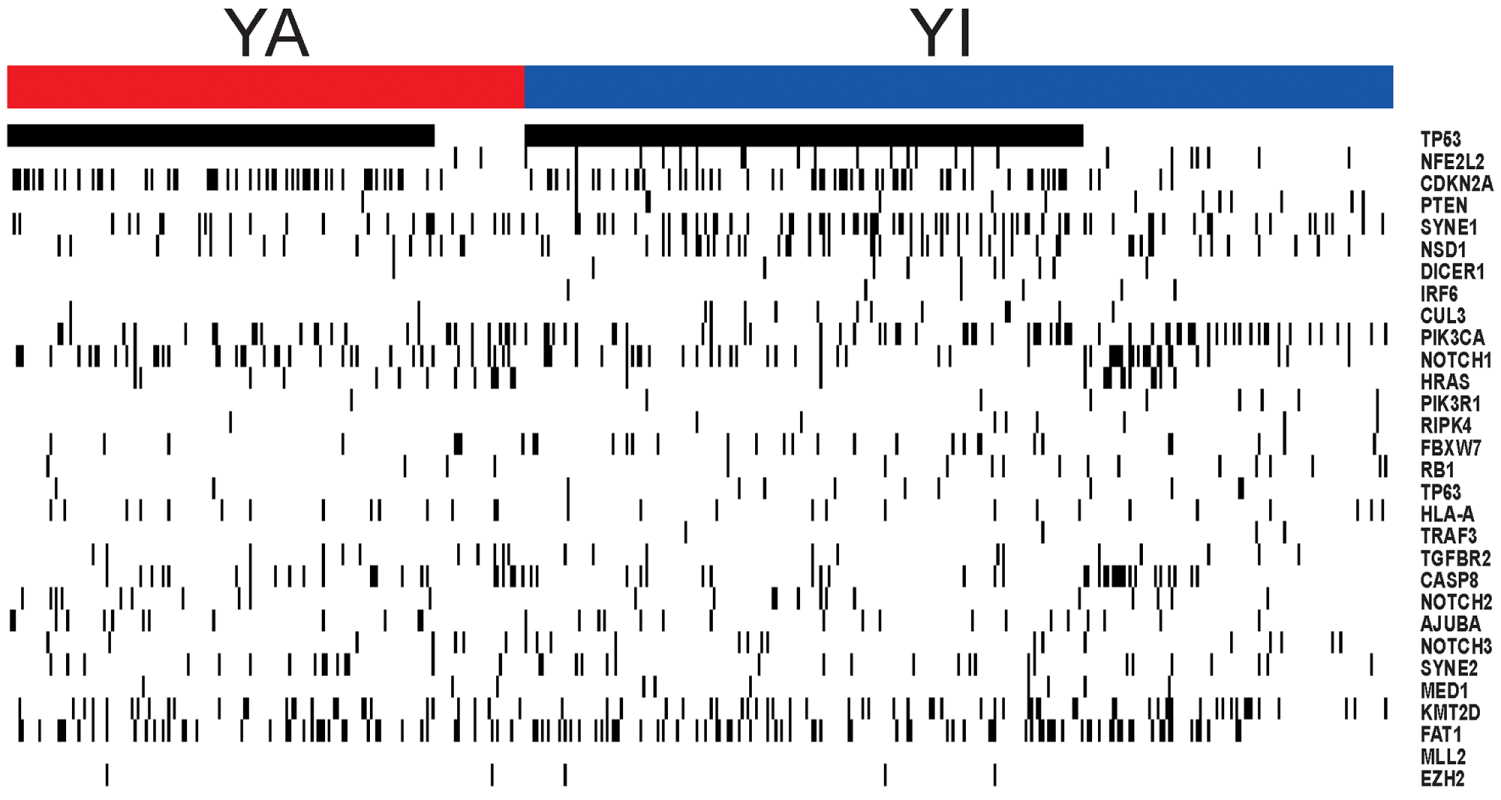
Somatic mutation in HNSCC according to two subgroups The mutations of TP53 and CDKN2A were identified as co-occurring mutated genes in YA subgroup. The mutations of NFE2L2, PTEN, SYNE1, and NSD1 were mutually exclusive for YAP1 activation.

To assess co-occurring somatic mutations with YAP1 activation in HNSCC, we also analyzed somatic mutation data in TCGA cohort (n = 493). When 30 significantly mutated genes in HNSCC were analyzed for association with YAP1 activation [13, 27], TP53 (82.6% vs. 64.4%, P = 1.4 × 10^-5^) and CDKN2A (28.3% vs. 19.1%, P = 0.02) were identified as co-occurring mutated genes (Figure 4, Supplementary Table 3). The mutations of NFE2L2 (1.1% vs. 7.8%, P = 7.0 × 10^-4^), PTEN (0.5% vs. 4.2%, P = 0.022), SYNE1 (14.1% vs. 22.3%, P = 0.025), and NSD1 (8.2% vs. 14.9%, P = 0.033) were mutually exclusive for YAP1 activation. Interestingly, AJUBA has been implicated in the Hippo pathway and was segregated predominantly in HPV-negative tumors [27]. However, AJUBA did not significantly differ between groups (7.1% vs. 5.8%, P = 0.57).

### miRNAs associated with YAP1 activation

**Figure 5:**
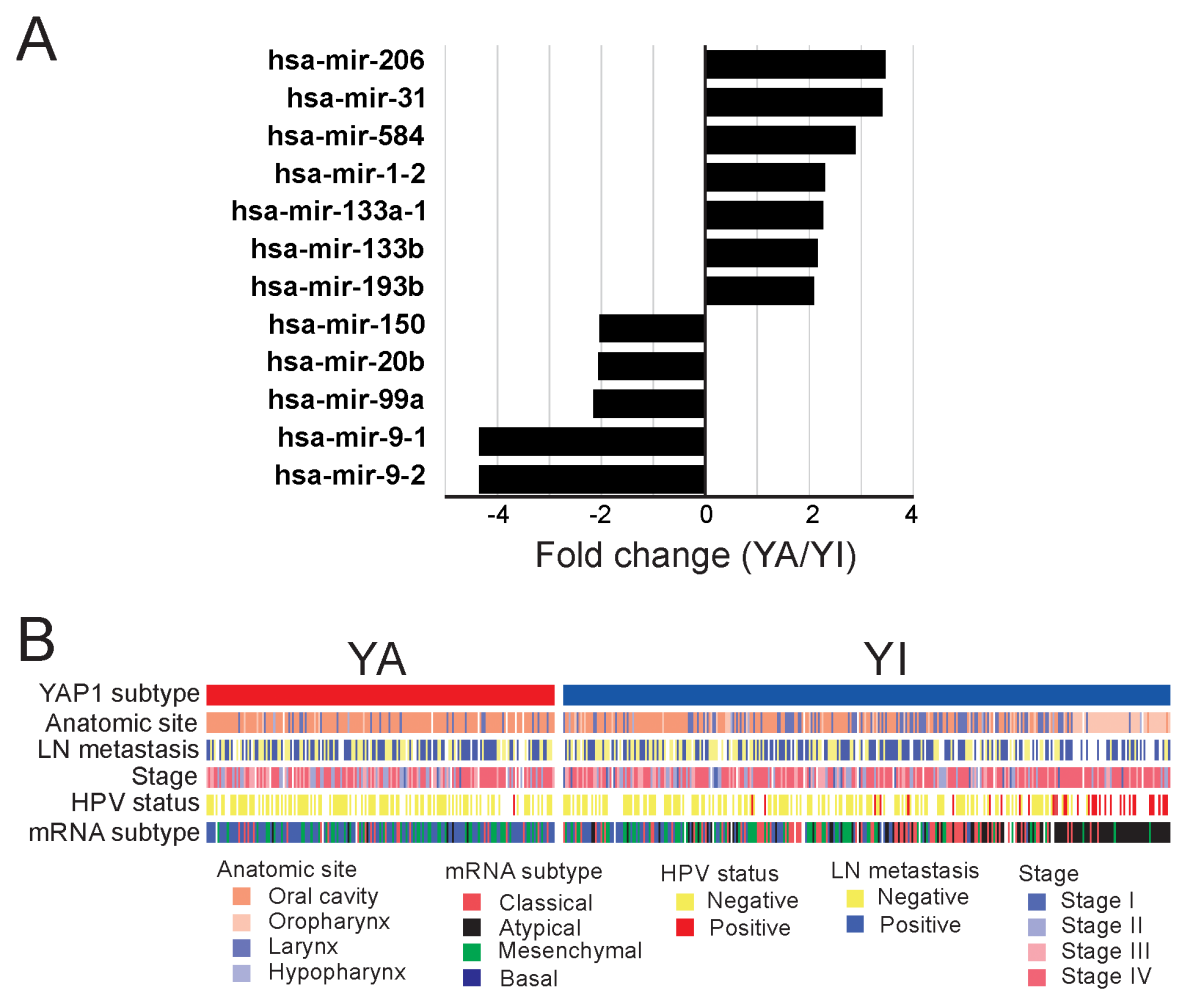
Molecular characteristics of the YA and YI subtypes **(A)** miRNAs that are differentially expressed in the YA and YI subtypes. **(B)** Association between YAP1 signature and the other subtypes of HNSCC. HPV infection status was most significantly associated with the two subtypes. Of 105 YA patients, 104 were HPV-negative (P = 4.29 × 10^-7^).

To explore potential interactions between YAP1 activation and miRNAs in HNSCC, we analyzed miRNA expression data from TCGA cohort (n = 473 tumors). Expression of 12 miRNAs was significantly associated with YAP1 activation (fold change >2, P < 0.001) (Figure 5A, Supplementary Table 4). miRNA-1-2, miRNA-133a, miRNA-133b, and miRNA-206 are known to be frequently downregulated in cancer [22]. They are also known to be involved in the regulation of molecular networks such as Wnt signaling pathway, tight junction, and MAPK signaling pathway in cancer [22]. Because these miRNAs were significantly upregulated in the YA subtype, they might be regulated through YAP1-associated signaling pathways.

Because many tumors in the YA subtype had high expression of YAP1 without YAP1 amplification (Figure 1A), we hypothesized that certain miRNAs might play roles in the regulation of YAP1 in these tumors [copy number-independent (CNI)-YA]. Thus, we sought to find miRNAs whose expression is specifically higher in CNI-YA than in copy number-dependent (CND) YA, YI, or normal head and neck tissues. We first selected miRNAs whose expression significantly differed between CNI-YA and CND-YA (fold ratio >1.5, P < 0.01) and further selected miRNAs whose expression was significantly different between CNI-YA and normal head and neck tissues (P < 0.05), yielding 21 miRNAs (Supplementary Table 5). Of these miRNAs, only expressions of miRNA-187 and miRNA-675 were significantly higher in CNI-YA than in YI or in normal tissues (Supplementary Figure 4). In good agreement with our hypothesis, many of the predicted target genes of both miRNAs are negative upstream regulators of YAP1 in the Hippo pathway (Supplementary Table 6, Supplementary Figure 5), suggesting that YAP1 in CNI-YA tumors might be activated through miRNA-mediated mechanisms.

### Clinical characteristics associated with YAP1 activation

We next assessed the association of the two subtypes with clinical characteristics of HNSCC in TCGA cohort. Among clinically recognized features such as lymph-node metastasis and T stages, YAP1 activation was significantly associated with HPV status (P = 4.29 × 10^-7^, Figure 5B). In TCGA cohort, HPV status data were available from only 279 of the 513 patients. Of 36 HPV-positive patients, most (n = 35) were in the YI subgroup. We also assessed the association of YAP1 activation with four previously recognized molecular subtypes of HNSCC (Supplementary Table 7) [28]. YAP1 activation was most associated with the basal subtype, as reflected in the fact that the highest proportion of patients (58.7%) in the YA subtype was from the basal subtype (P = 2.2 × 10^-6^, Figure 5B). TheYI subgroup lacked any significant association with the four subtypes as it was found in similar proportions in the four subtypes: atypical subtype (33.3%), classical subtype (24.9%), mesenchymal subtype (22.0%), and basal subtype (19.7%).

### Association of YAP1 activity with host immune activity

**Figure 6:**
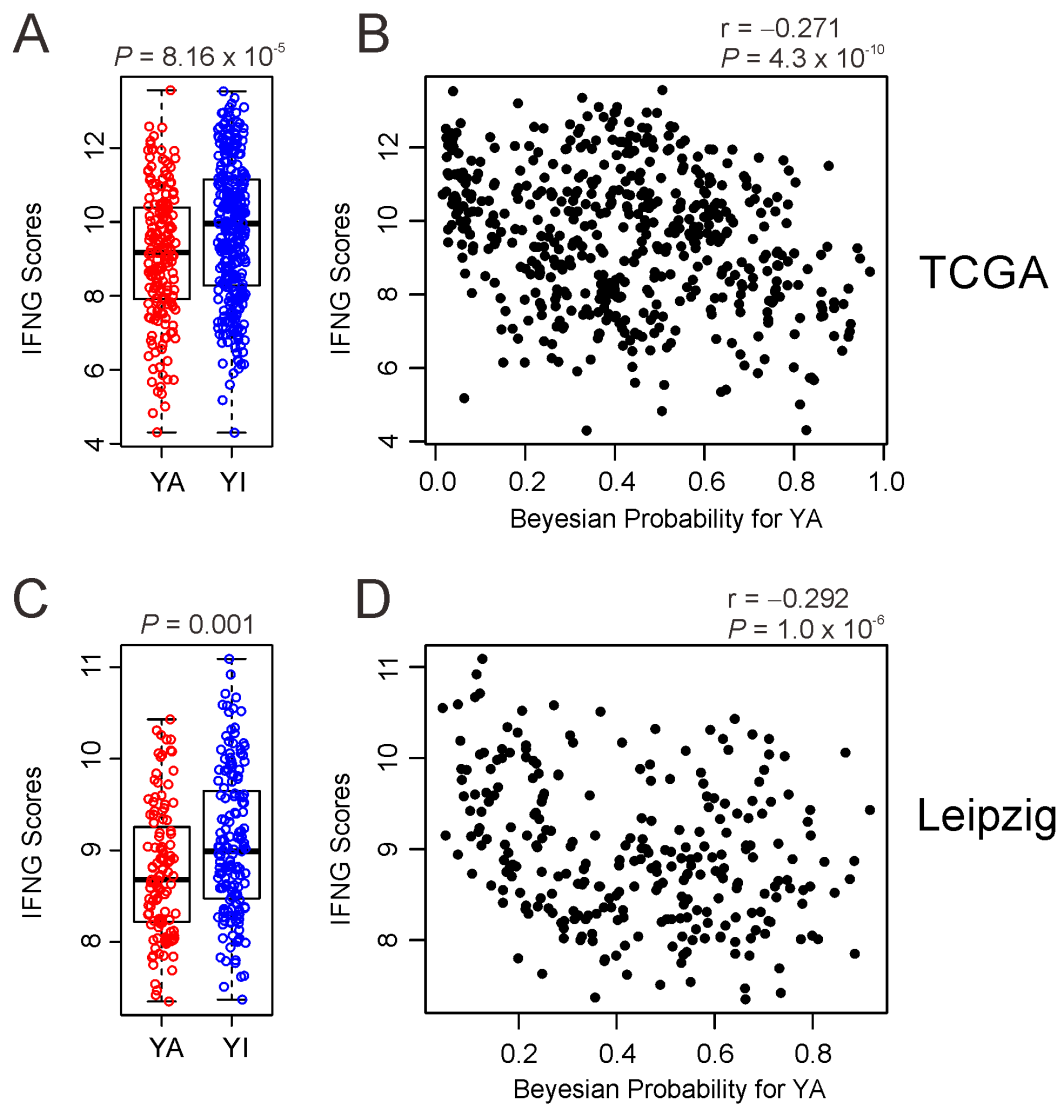
Negative association of YAP1 activity with IFNG scores in HNSCC **(A, C).** Comparison of the IFNG scores between YA subgroup and YI subgroup in TCGA and Leipzig cohort. **(B, D)** Scatter plots between IFNG scores and Bayesian probability of YAP1 activity is TCGA and Leipzig cohort.

Recent clinical trial demonstrated clinically meaningful anti-tumor activity of pembrolizumab, humanized antibody against immune checkpoint inhibitor PD-1, in HNSCC [29]. Since study also showed that composite scores from expression of 6 interferon γ (INFG)-related genes (CXCL9, CXCL10, IDO1, IFNG, HLA-DRA, and STAT1) were good predictor for identifying responders of pembrolizumab treatment, we next assessed association of two subtypes with potential response to permbrolizumab treatment by generating INFG composite scores in TCGA cohort. Interestingly, YA subtype has significantly low INFG composite scores (*P* =8.1 x 10^-5^) (Figure 6A), suggesting that YAP1 may suppress immune activity related immune checkpoint regulation. Further support of the idea is supplied by significant negative correlation between IFNG composite scores and Bayesian probability of active YAP1 in TCGA cohort (Figure 6B). Consistent with results from TCGA cohort, IFNG scores were significantly lower in YA subtype (*P* = 0.001) and negatively correlated with Bayesian probability of active YAP1 in Leipzig cohort (Figure 6C and 6D).

## DISCUSSION

By systematically analyzing genomic copy number data and mRNA expression data of YAP1 in HNSCC, we identified a gene expression signature reflecting YAP1 activation (YAP1 signature) that is significantly associated with the prognosis of patients with HNSCC. Robustness of the signature was validated in five independent cohorts of patients with HNSCC, as patients with the YA subtype had worse survival rates than patients with the YI subtype in all examined cohorts. The YAP1 signature was an independent prognostic factor. In multiplatform analysis, YA patients had gain of EGFR and SNAI2; loss of tumor-suppressor genes such as CSMD1, CDKN2A, NOTCH1, and SMAD4; and high mutation rates of TP53 and CDKN2A. YI patients were characterized by gain of PIK3CA, SOX2, and TP63; deletion of 11q23.1; and high mutation rates of NFE2L2, PTEN, SYNE1, and NSD1. To our knowledge, we are the first to report the clinical significance of YAP1 activation in HNSCC and demonstrate that the YAP1 signature can be used as a prognostic biomarker for HNSCC.

Our study results are in good agreement with previous observations in other cancers [18, 30-34]. Activation of YAP1 has been correlated with poor prognosis for colorectal cancer and poor response to cetuximab [18]. Likewise, activation of YAP1 was significantly associated with poor prognosis in ovarian cancer, liver cancer, and gastric cancers [18, 32-34]. Previous studies showed that YAP1 had a role in the onset, progression, and drug resistance in HNSCC [14, 15, 19, 20]. Hiemer et al. reported that YAP1 and TAZ activity drives oral squamous cell carcinoma proliferation, survival, and migration *in vitro* and vivo [14]. YAP expression was elevated in tumor compared with benign tissues and was associated with nodal metastasis [15]. A potential association of YAP1 with resistance to radiation therapy was also supported by previous studies, as amplification of YAP1 was correlated with cetuximab sensitivity in HNSCC [20] and knockdown of YAP1 increased the sensitivity to cisplatin *in vitro* [19].

Comparisons of copy number alteration and somatic mutation showed significant differences between the two subtypes. The YA group had more loss of copy number and a higher somatic mutation rate of the cell cycle–related gene CDKN2A. In good agreement with a previous study demonstrating that deletion of CDKN2A and inactivating mutation were associated with HPV-negative tumors [27], the vast majority of tumors with the YA subtype were HPV-negative tumors, suggesting that YAP1 activation and inactivation of CDKN2A might be associated genetic events. Thus, it will be interesting to determine in future investigations whether YAP1 can downregulate CDKN2A. Tumors of the YA subtype were also characterized by gain of EGFR and SNAI2. SNAI2 has five zinc finger domains that play a pivotal role during embryo development and mesenchymal tumorigenesis and has been found to be overexpressed in several cancers, and it also promotes invasion in lung adenocarcinoma, glioma, and ovarian, cervical, and pancreatic cancers and is a prognostic marker in some cancers [24, 35]. The YA subtype was also characterized by a higher mutation rate of the tumor-suppressor gene associated with cell survival (TP53) and loss of copy number of tumor-suppressor genes (CSMD1, NOTCH1, and SMAD4), while the YI subtype was characterized by high mutation rates of NFE2L2, PTEN, SYNE1, and NSD1. Spectrin repeat containing nuclear envelope protein 1 (SYNE1) gene has been implicated in the regulation of nuclear polarity, a process that operates upstream of NOTCH1 in squamous epithelia [35]. SYNE1 mutation was associated with autosomal recessive cerebellar ataxia [24] and is known to be associated with glioblastoma and lung, ovarian, colorectal, and head and neck cancers [35, 36]. The nuclear receptor binding SET domain protein 1 (NSD1), a histone methyltransferase, was found to be frequently mutated in the clear cell variant of renal cell carcinoma and associated with DNA hypomethylation [27].

In assessing the association of the YAP1 subgroup with the four previously discovered molecular subtypes [28], we found that the YA group had the most similarity with the basal subtype. The basal subtype was characterized by inactivation of NOTCH1 and co-amplified 11q13/q22 (21) in which YAP1 resided. Interestingly, 94.5% of atypical subtype was YI subtype (Supplementary Table 7). The atypical subtype was characterized by enrichment of HPV-positive tumor and activating mutations in exon 9 that contain PIK3CA (21). In good agreement with this, the YI subtype included most of the HPV-positive tumors and featured the amplification of copy number of PIK3CA. While the YAP1-associated subtype shares some molecular features with the four previously recognized molecular subtypes, it is important to point out that this subtype is clinically relevant as reflected in prognostic difference and response to radiation therapy, while clinical association of the four molecular subtypes has not been clearly demonstrated yet [28].

Recent advances in our understanding of onco-immunology has led to the development of immunotherapies. Particularly, a blockade of checkpoint molecules such as CTLA-4 and PD-1 has emerged as a novel therapeutic approach in oncology [37-39]. PD-1 is a negative co-stimulatory receptor and a strong inhibitor of T cell response [40]. Pembrolizumab targeting PD-1 has been approved for the treatment of multiple cancers including metastatic melanoma [41]. Six-gene based IFNG composite scores were developed and tested as predictor for response to pembrolizumab treatment in HNSCC [29]. When immune activity reflecting potential response to pembrolizumab in HNSCC tumors was assessed by using IFNG scores, tumors in YA subtype have lower scores and IFNG scores were negatively correlated with YAP1 activity, suggesting that YAP1 may be involved in regulation of host immunity against cancer cells. Thus, YA subtype might be more resistant to immunotherapy. Since YA subtype is poor prognostic, it would be also interested to see if YAP1-mediated low immune activity contributes to aggressiveness of cancer cells in YA subtype in future study.

This study has some limitations. First, all cohorts examined in our study were retrospective cohorts. Second, while patients in the YI subgroup showed better survival than those in the YA subgroup when treated with radiation therapy, an interaction test failed to show significant interaction between subgroups and radiation therapy. Thus, these observations should be validated in future prospective study.

In conclusion, our newly discovered clinically relevant subgroup may help facilitate rational design of clinical studies by stratifying patients according to their prognostic risk and response to therapy. Prospective studies are needed to validate the clinical usefulness of the two subgroups.

## MATERIALS AND METHODS

### Patients and gene expression data

This study used patient data from five independent sources. Gene expression, mutation, miRNA, and copy number data of The Cancer Genome Atlas (TCGA) cohort were downloaded from the UCSC Cancer Genomics Browser (https://genome-cancer.ucsc.edu/) [27]. mRNA expression, miRNA expression, copy number alteration, and mutation data were available from 513, 473, 513, and 493 patients respectively. We also used the gene expression and clinical data for four independent cohorts available from the National Center for Biotechnology Information (NCBI) Gene Expression Omnibus (GEO) database (http://www.ncbi.nlm.nih.gov/geo). These four cohorts consisted of data from the Institute for Medical Informatics, Statistics and Epidemiology (Leipzig cohort, GSE65858, n = 270) [42], Aristotle University of Thessaloniki (Greek cohort, GSE27020, n = 109) [43], MD Anderson Cancer Center (MD Anderson cohort, GSE42743, n = 74) [44], and Fred Hutchinson Cancer Research Center (Seattle cohort, GSE41613, n = 97) [44]. Table 1 shows the pathologic and clinical characteristics of the patients in all five cohorts.

### Statistical analysis

The BRB-ArrayTools software program (http://brb.nci.nih.gov/BRB-ArrayTools/) was used for analysis of gene expression data [45]. The R language environment (http://www.r-project.org) was used for other statistical analyses. Raw data on the patient cohorts were normalized using a robust multiarray averaging method [46]. A stringent threshold was used to minimize the number of false-positive findings. Pearson correlation was used for correlation analysis. We estimated prognoses using Kaplan-Meier plots and the log-rank test. We used univariate and multivariate Cox proportional hazards regression analyses to evaluate independent prognostic factors associated with survival. Independent *t*-test was used to compare values between two groups. Fisher’s exact test was used to evaluate the frequency difference of copy number alteration and somatic mutation. P values less than 0.05 indicated statistical significance, and all statistical tests were two-tailed. A heatmap was generated using the Cluster and TreeView software programs [47]. Target genes of miRNAs were predicted by using miRWalk 2.0 (http://zmf.umm.uni-heidelberg.de/) [48].

### Gene expression data and construction of the prediction model

Gene expression data of TCGA cohort were sequenced by Illumina HiSeq2000, the Leipzig cohort by Illumina HumanHT-12 V4.0 expression beadchip, the Greek cohort by Affymetrix Human Genome U133A Array, and the MD Anderson and Seattle cohorts by Affymetrix Human Genome U133 Plus 2.0 Array. All gene expression data were standardized independently across all samples before they were integrated and analyzed together. The strategy used to develop and validate the prediction model on the basis of the gene expression signature and to estimate predictive accuracy was adopted from previous studies [49-51]. To find YAP1-specific genes in HNSCC, we applied a double correlation approach to gene expression data from TCGA cohort. We first identified YAP1-associated genes by using the correlation between copy number of YAP1 and mRNA expression of each gene (copy number-associated genes). Likewise, we also identified second YAP1-associated genes by using the correlation between mRNA expression of YAP1 and mRNA expression of each gene (mRNA-associated genes). Genes were selected if the P value was less than 0.001 and the correlation coefficient was more than 0.2 or less than –0.2. Expression patterns of 292 shared genes in two YAP1-associated gene lists were considered as the YAP1 signature in HNSCC and used for construction of the prediction model. We used data from TCGA cohort as the training set and data from the Leipzig, Greek, MD Anderson, and Seattle cohorts as test sets. The expression patterns of the 292 genes from TCGA cohort were combined to form a classifier according to a Bayesian compound covariate (BCCP) predictor [52]. The robustness of the classifier was estimated using a misclassification rate determined during leave-one-out cross-validation in the training set. The BCCP classifier estimated the likelihood that an individual patient had either a YAP1-active subtype or YAP1-inactive subtype.

### Copy number analysis

We used the HNSCC copy number data (gistic2) in Cancer Browser (https://genome-cancer.ucsc.edu). Threshold copy number at reoccurring alteration peaks from GISTIC analysis of BRB CGH-Tools was used for comparison of focal peak frequency across subgroups by YAP1 signature. Fisher’s exact test was used for frequency comparisons of significantly reoccurring alterations by YAP1 signature. For comparison of amplification, only high-level events were considered (gistic ≥2), and for deletion all events were used as described in a previous study (21).

### Association with YAP1 signature and other subtypes of HNSCC

For assessing the association with previously published molecular classifications of HNSCC [28], we evaluated four previously established molecular classifications: atypical, classical, basal, and mesenchymal. The predictor was adjusted from 790 genes. Each sample was assigned an expression subtype using the BCCP predictor.

### Association with YAP1 signature and interferon interferon γ signature

A six-gene signature of interferon γ-related genes (CXCL9, CXCL10, IDO1, IFNG, HLA-DRA, and STAT1) was used in TCGA cohort. The interferon γ score was calculated as the average of the value of the six genes. The interferon γ score was compared with *t*-test between YA group and YI group.

## SUPPLEMENTARY MATERIALS FIGURES AND TABLES










