# Supplementary material for: Clinical significance of YAP1 activation in head and neck squamous cell carcinoma
Source: Oncotarget. 2017 Nov 27;8(67):111130–43. doi: 10.18632/oncotarget.22666 (PMC5762311; doi:10.18632/oncotarget.22666)
Supplement: Supplementary file 1 [file oncotarget-08-111130-s001.pdf]

Clinical significance of YAP1 activation in head and neck squamous cell carcinoma

SUPPLEMENTARY MATERIALS

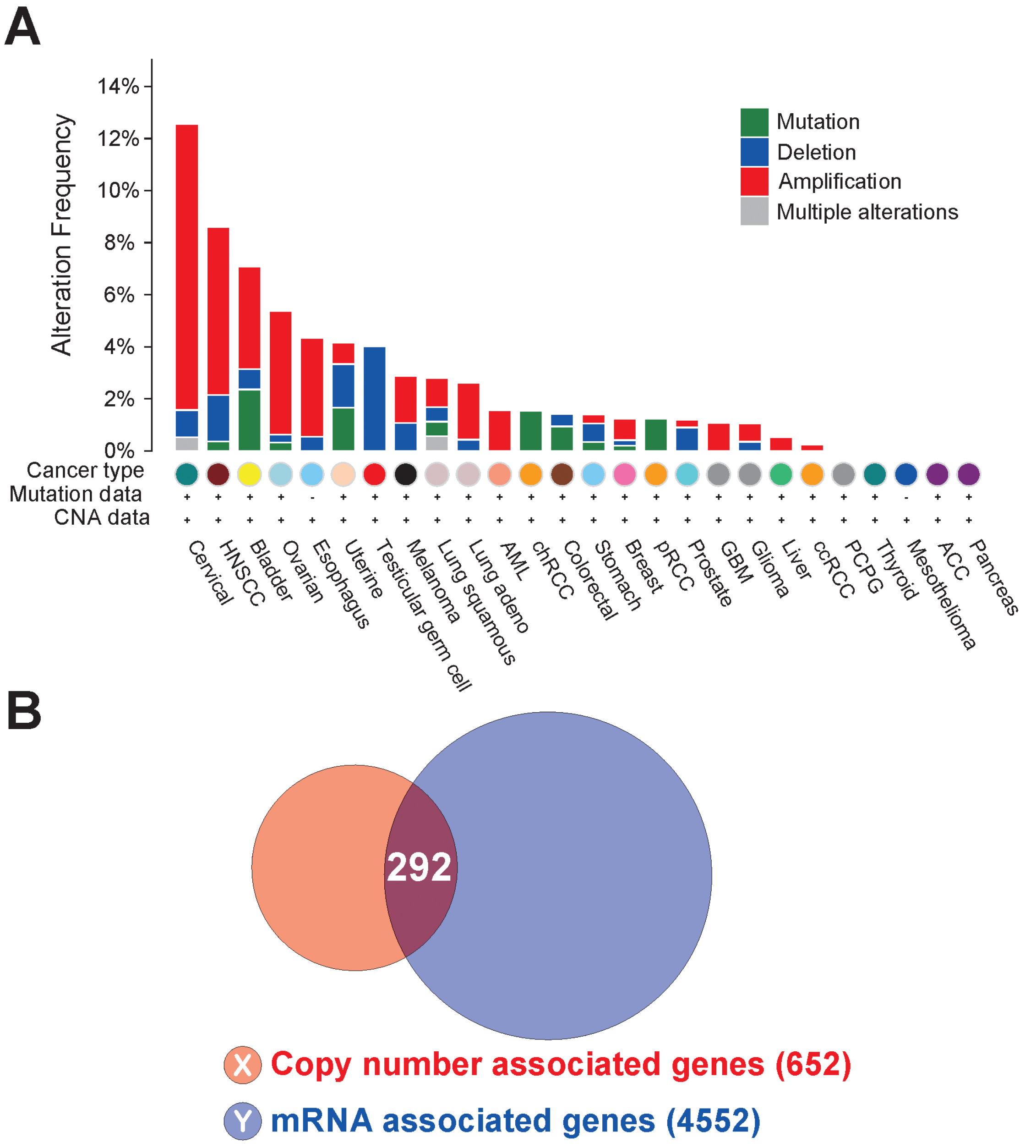

Supplementary Figure 1: Activation of YAP1 in HNSCC. (A) Cross-cancer alteration summary for YAP1 in 26 cancers by using genome copy number data from The Cancer Genome Atlas (TCGA) project. HNSCC is the second most YAP1-amplified cancer type (8.6%) after cervical cancer (12.6 %). (**B)** Venn diagram showing the genes (n=292) which are shared in copy number associated genes and mRNA associated genes. The red circle (gene list X) represents copy number associated genes whose expression is significantly correlated with copy number alterations ($P < 0.001$ and Pearson correlation coefficient > 0.2 or < -0.2). The blue circle (gene list Y) represents mRNA associated genes whose expression is significantly correlated with mRNA expression of YAP1 ($P < 0.001$ and Pearson correlation coefficient > 0.2 or < -0.2).

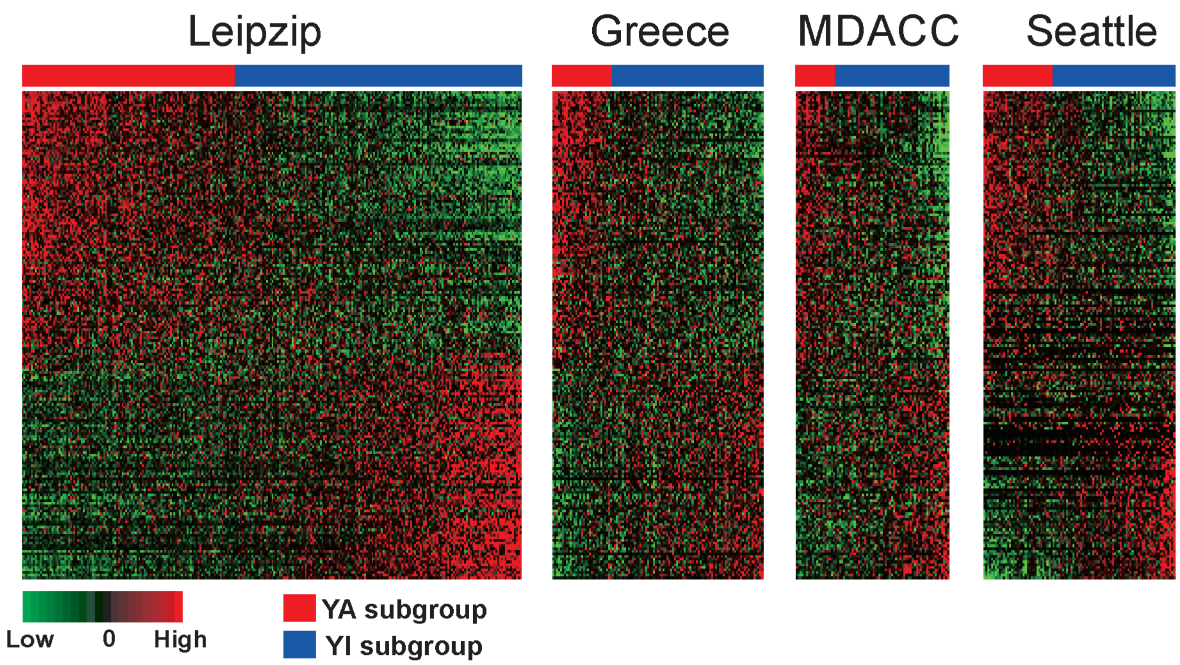

Supplementary Figure 2: Expression patterns of 292 genes in the YAP1 signature in four independent test cohorts of HNSCC patients.

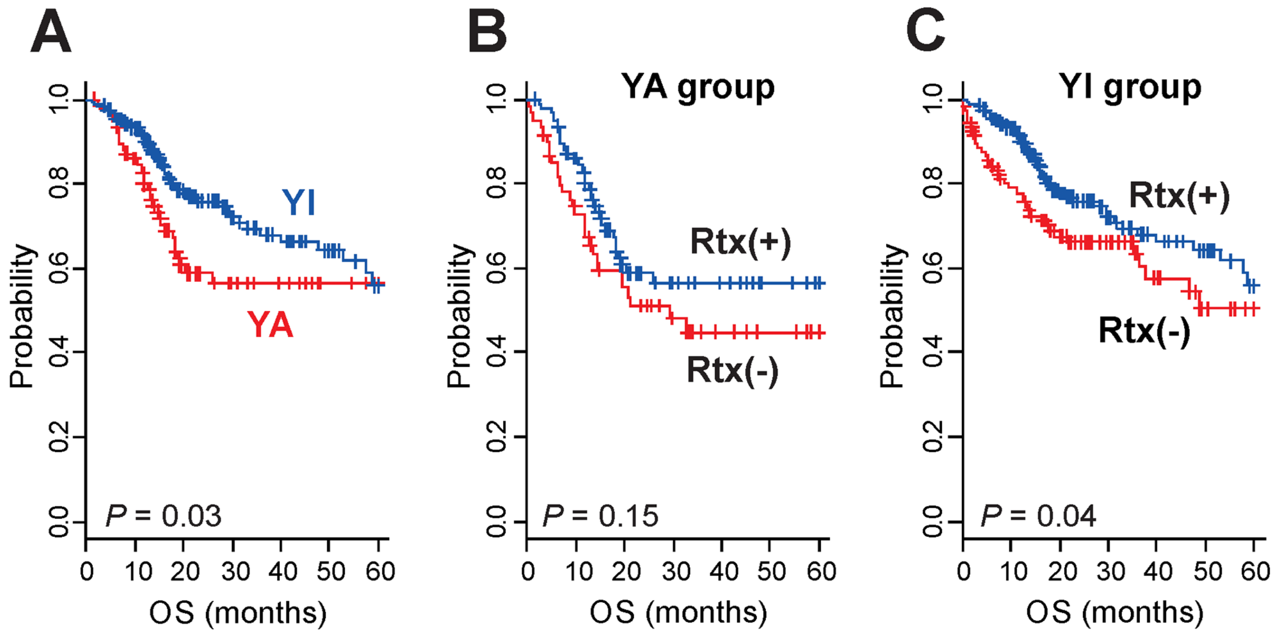

Supplementary Figure 3: YI subtype is more sensitive to radiation therapy. **(A)** Kaplan–Meier plots of the YA and YI patients with radiation therapy. For the patients who underwent radiation therapy, the survival of YA was significantly worse than YI. **(B)** Kaplan–Meier plots of the YA subtype across radiation therapy. For YA group, radiation therapy did not improve the survival rate. **(C)** Kaplan–Meier plots of the YI subtype across radiation therapy. Radiation therapy improved the survival rate of YI group. P values were calculated using log-rank tests. +, censored data.

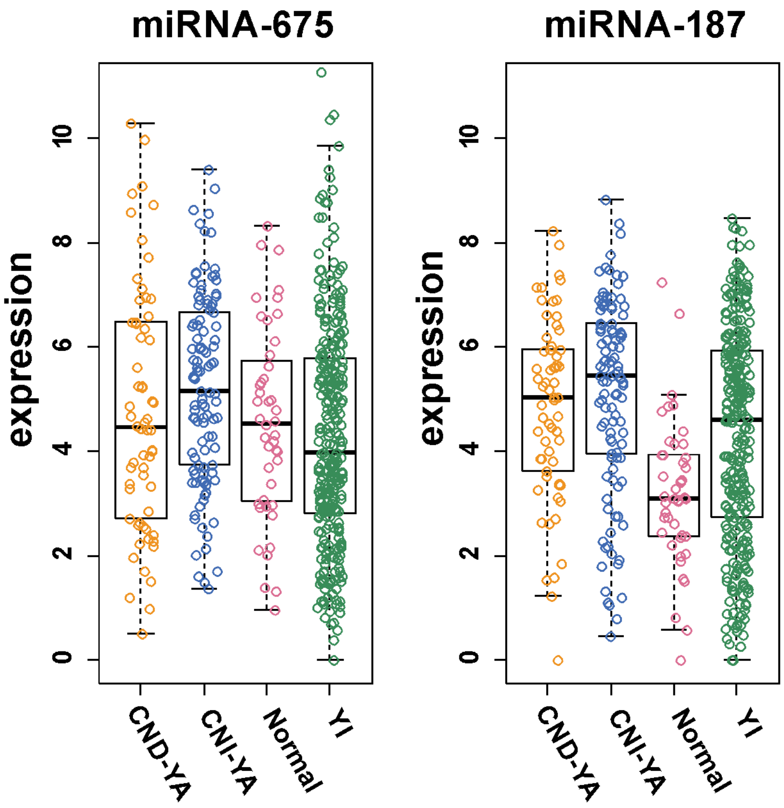

Supplementary Figure 4: Expression of miRNA-675 and miRNA-187 by YAP1 subtypes. Expression of miRNA-187 and miRNA-675 were significantly higher in CNI-YA than in CND-YA, YI and normal tissues. CND-YA: copy number dependent YAP1 activated tumors, CNI-YA: copy number independent YAP1 activated tumors, YI: YAP1 inactivated tumors.

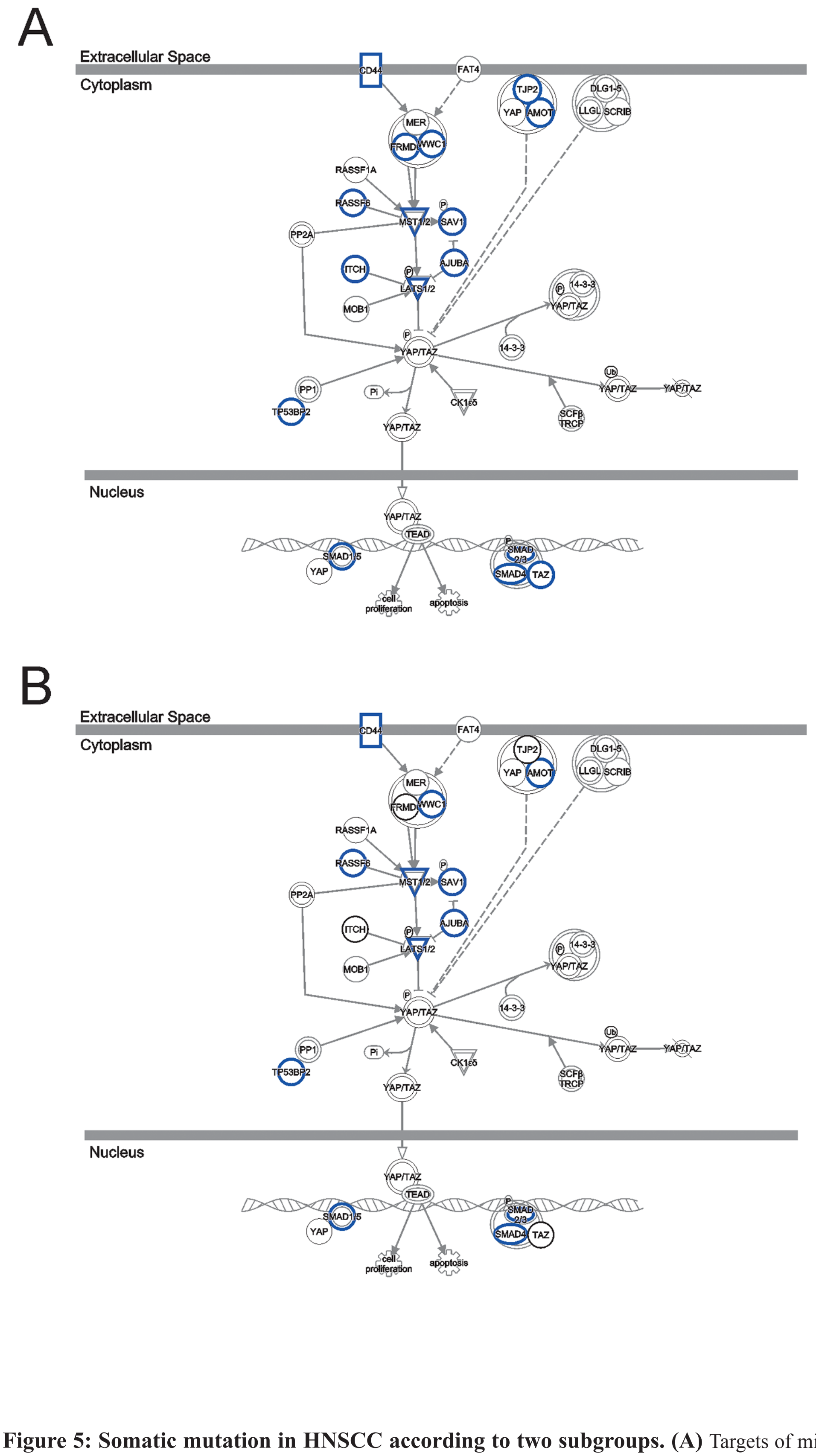

Supplementary Figure 5: Somatic mutation in HNSCC according to two subgroups. (A) Targets of miRNA-187 in Hippo pathway. **(B)** Targets of miRNA-675 in Hippo pathway.

Supplementary Table 1: Genes associated with YAP1.

See Supplementary File 1

Supplementary Table 2: Fisher's exact test p values for frequency comparisons of significantly reoccurring alterations by YAP1 subtypes.

See Supplementary File 2

Supplementary Table 3: Comparison of somatic mutation frequencies between YA and YI subtypes.

See Supplementary File 3

Supplementary Table 4: miRNAs that were signinficantly different between YA and YI subtypes

	Fold change	p.value
miRNA-206	3.463031099	4.87922E-06
miRNA-31	3.412275517	1.09814E-18
miRNA-584	2.889203427	4.48703E-32
miRNA-1-2	2.303772907	0.000114169
miRNA-133a-1	2.264936512	0.000140021
miRNA-133b	2.160290149	8.33789E-05
miRNA-193b	2.086079851	1.33671E-26
miRNA-150	-2.032934942	1.73115E-11
miRNA-20b	-2.06117528	5.10529E-11
miRNA-99a	-2.151538926	1.85804E-16
miRNA-9-1	-4.35004349	1.09271E-19
miRNA-9-2	-4.352154878	1.21538E-19

Supplementary Table 5: Comparison of miRNA expression of CNI-YA vs. CND-YA and YI or normal group

	Fold change of CNI-YA and others	p.value CNI-YA vs. others	p.value CNI-YA vs Normal
mir-133a-1	3.341	7.37502E-07	0.004771452
mir-1-2	3.203	2.18248E-06	0.000145416
mir-133b	2.930	1.34612E-06	0.003024693
mir-31	2.518	1.33178E-08	1.86616E-20
mir-133a-2	2.247	2.23402E-06	0.002172704
mir-584	2.222	9.26644E-14	2.29962E-12
mir-1293	1.989	1.24125E-08	3.84535E-21
mir-193b	1.855	2.17152E-14	2.84828E-36
mir-675	1.750	0.000466098	0.048341647
mir-451	1.634	5.72132E-05	0.017462427
mir-187	1.593	0.001843344	1.31498E-08
mir-455	1.572	1.13499E-08	1.61931E-41
mir-144	1.555	0.000217149	0.047859734
mir-503	1.551	8.64555E-08	9.00033E-34
mir-365-1	1.519	4.89091E-10	1.57912E-09
mir-196b	-1.503	0.00041557	8.11362E-28
mir-10a	-1.510	6.87762E-05	0.001524434
let-7c	-1.615	1.00874E-06	3.87041E-31
mir-125b-2	-1.633	5.07236E-06	7.87197E-22
mir-1266	-1.633	3.58824E-08	3.92939E-08
mir-29c	-1.653	1.30659E-07	2.88312E-29
mir-99a	-1.798	6.0809E-08	5.29194E-37

Supplementary Table 6: Predicted gene targets in Hippo signaling pathway

Predicted genes of miRNA-187		Predicted genes of miRNA-675	
**Gene**	**EntrezID**	**Gene**	**EntrezID**
CD44	960	CD44	960
SMAD1	4086	SMAD2	4087
SMAD2	4087	SMAD3	4088
SMAD3	4088	SMAD4	4089
SMAD4	4089	SMAD5	4090
SMAD5	4090	MST1	4485
MST1	4485	TAZ	6901
TAZ	6901	TP53BP2	7159
TP53BP2	7159	LATS1	9113
LATS1	9113	TJP2	9414
TJP2	9414	INADL	10207
INADL	10207	WWC1	23286
WWC1	23286	LATS2	26524
CRB1	23418	PARD3	56288
LATS2	26524	SAV1	60485
PARD3	56288	AJUBA	84962
SAV1	60485	AMOT	154796
ITCH	83737	RASSF6	166824
AJUBA	84962		
FRMD6	122786		
AMOT	154796		
RASSF6	166824		

Supplementary Table 7: Molecular classification and YAP1 subtype.

See Supplementary File 4
