# Supplementary material for: Clinical significance of YAP1 activation in head and neck squamous cell carcinoma
Source: Oncotarget. 2017 Nov 27;8(67):111130–43. doi: 10.18632/oncotarget.22666 (PMC5762311; doi:10.18632/oncotarget.22666)
Supplement: Supplementary file 2 [file oncotarget-08-111130-s002.docx]

| **Supplementary Table 1. Genes associated with YAP1** | | | |  |
| --- | --- | --- | --- | --- |
|  |  |  |  |  |
| TCGA RNA sequencing gene symbol | correlation coefficient  with copy number of YAP1 | p value of correlation  with copy number of YAP1 | correlation coefficient  with gene expression of YAP1 | p value of correlation  with gene expression of YAP1 |
| ABCA17P | -0.218 | 6.06712E-07 | -0.345 | 9.30472E-16 |
| ABCA3 | -0.205 | 2.83366E-06 | -0.276 | 1.99357E-10 |
| ACACB | -0.202 | 3.8934E-06 | -0.213 | 1.16352E-06 |
| ACER3 | 0.220 | 4.6242E-07 | 0.373 | 2.03354E-18 |
| ACO2 | -0.201 | 4.70954E-06 | -0.257 | 3.31153E-09 |
| ACSM3 | -0.206 | 2.48869E-06 | -0.207 | 2.18864E-06 |
| ACTBL2 | 0.204 | 3.33592E-06 | 0.275 | 2.21183E-10 |
| ACTN1 | 0.226 | 2.20182E-07 | 0.365 | 1.16511E-17 |
| ADAMTSL4 | 0.276 | 2.04782E-10 | 0.320 | 1.04845E-13 |
| ADAMTSL5 | 0.250 | 9.39111E-09 | 0.349 | 4.07062E-16 |
| ADARB2 | -0.213 | 1.15425E-06 | -0.214 | 1.04215E-06 |
| AGPAT4 | 0.205 | 2.84811E-06 | 0.228 | 1.88617E-07 |
| ALG8 | 0.247 | 1.37379E-08 | 0.227 | 2.08513E-07 |
| ALPP | 0.302 | 2.72475E-12 | 0.298 | 5.31412E-12 |
| AMOTL1 | 0.260 | 2.22811E-09 | 0.533 | 4.8536E-39 |
| AMOTL2 | 0.207 | 2.28415E-06 | 0.450 | 5.64398E-27 |
| ANKRD49 | 0.223 | 3.517E-07 | 0.285 | 4.47967E-11 |
| ANXA3 | 0.202 | 4.06517E-06 | 0.283 | 6.89683E-11 |
| APBB2 | 0.205 | 2.93752E-06 | 0.364 | 1.7016E-17 |
| APCDD1L | 0.243 | 2.39326E-08 | 0.333 | 8.61875E-15 |
| APOBEC3D | -0.229 | 1.55151E-07 | -0.271 | 4.04318E-10 |
| APOBEC3G | -0.208 | 1.92926E-06 | -0.295 | 8.53848E-12 |
| APOBEC3H | -0.206 | 2.40215E-06 | -0.350 | 3.36938E-16 |
| ARAP1 | 0.368 | 6.30256E-18 | 0.261 | 1.98242E-09 |
| ARHGAP42 | 0.346 | 7.61533E-16 | 0.489 | 2.90241E-32 |
| ARHGEF17 | 0.247 | 1.47536E-08 | 0.323 | 5.80737E-14 |
| ASRGL1 | -0.245 | 1.88582E-08 | -0.349 | 4.15966E-16 |
| BASP1 | 0.221 | 4.53011E-07 | 0.213 | 1.10439E-06 |
| BCAR1 | 0.261 | 2.07672E-09 | 0.244 | 2.25734E-08 |
| BCL11A | -0.203 | 3.72075E-06 | -0.217 | 6.98931E-07 |
| BCL2 | -0.259 | 2.77253E-09 | -0.215 | 8.6505E-07 |
| BCL2L14 | -0.224 | 2.78792E-07 | -0.263 | 1.55043E-09 |
| BDH1 | -0.204 | 3.01229E-06 | -0.223 | 3.5035E-07 |
| BIRC2 | 0.835 | 4.0592E-135 | 0.833 | 1.1076E-133 |
| BIRC3 | 0.281 | 9.12946E-11 | 0.257 | 3.27733E-09 |
| BTNL9 | -0.231 | 1.23504E-07 | -0.285 | 4.69371E-11 |
| C11orf70 | 0.421 | 1.60919E-23 | 0.302 | 2.60078E-12 |
| C11orf85 | -0.225 | 2.47242E-07 | -0.300 | 4.24515E-12 |
| C11orf92 | -0.219 | 5.45666E-07 | -0.255 | 4.69684E-09 |
| C11orf93 | -0.219 | 5.56633E-07 | -0.232 | 1.00237E-07 |
| C12orf34 | -0.239 | 3.99205E-08 | -0.241 | 3.06723E-08 |
| C13orf29 | 0.221 | 4.142E-07 | 0.298 | 5.88237E-12 |
| C18orf55 | -0.257 | 3.35104E-09 | -0.286 | 3.87909E-11 |
| C19orf57 | -0.207 | 2.2188E-06 | -0.325 | 4.59793E-14 |
| C20orf132 | -0.208 | 2.11093E-06 | -0.329 | 2.04025E-14 |
| C22orf31 | -0.249 | 1.13521E-08 | -0.289 | 2.50883E-11 |
| C3orf42 | -0.207 | 2.38349E-06 | -0.253 | 5.81897E-09 |
| C6orf218 | 0.245 | 1.98612E-08 | 0.273 | 3.39227E-10 |
| C9orf45 | -0.230 | 1.40349E-07 | -0.265 | 1.12274E-09 |
| CA11 | -0.209 | 1.75455E-06 | -0.370 | 4.43312E-18 |
| CACNA1B | -0.206 | 2.65321E-06 | -0.260 | 2.36326E-09 |
| CASP14 | 0.202 | 4.21657E-06 | 0.352 | 2.15924E-16 |
| CAV1 | 0.232 | 1.00634E-07 | 0.334 | 7.55026E-15 |
| CAV2 | 0.208 | 1.9152E-06 | 0.354 | 1.32615E-16 |
| CBX7 | -0.280 | 1.0336E-10 | -0.288 | 2.84808E-11 |
| CCBE1 | 0.214 | 9.52545E-07 | 0.255 | 4.44052E-09 |
| CCDC155 | -0.210 | 1.58332E-06 | -0.293 | 1.28618E-11 |
| CCDC160 | -0.215 | 9.12874E-07 | -0.251 | 8.61469E-09 |
| CCDC64 | -0.234 | 7.75657E-08 | -0.217 | 7.10214E-07 |
| CCDC90B | 0.271 | 4.30479E-10 | 0.219 | 5.35872E-07 |
| CD2 | -0.203 | 3.40549E-06 | -0.270 | 4.80059E-10 |
| CD6 | -0.207 | 2.30154E-06 | -0.288 | 2.82436E-11 |
| CD8A | -0.224 | 2.84685E-07 | -0.261 | 1.95031E-09 |
| CD8B | -0.243 | 2.32409E-08 | -0.315 | 2.92187E-13 |
| CDA | 0.203 | 3.71071E-06 | 0.227 | 2.0563E-07 |
| CHAF1B | -0.210 | 1.58626E-06 | -0.306 | 1.45752E-12 |
| CHORDC1 | 0.264 | 1.24563E-09 | 0.373 | 2.14241E-18 |
| CHPF2 | 0.231 | 1.27411E-07 | 0.218 | 6.18414E-07 |
| CHPT1 | -0.201 | 4.36117E-06 | -0.248 | 1.25825E-08 |
| CLDN10 | -0.245 | 1.78479E-08 | -0.263 | 1.39303E-09 |
| CLDN3 | -0.208 | 1.90263E-06 | -0.260 | 2.11864E-09 |
| CLGN | -0.218 | 6.02831E-07 | -0.275 | 2.2359E-10 |
| CLIC6 | -0.269 | 6.06007E-10 | -0.216 | 7.89175E-07 |
| CLPB | 0.318 | 1.5535E-13 | 0.267 | 8.53278E-10 |
| CNTN5 | 0.236 | 6.21875E-08 | 0.208 | 1.9251E-06 |
| COL4A6 | 0.238 | 4.61752E-08 | 0.367 | 7.48382E-18 |
| CPA4 | 0.232 | 1.10148E-07 | 0.329 | 2.05634E-14 |
| CRB2 | -0.220 | 4.91445E-07 | -0.225 | 2.52626E-07 |
| CRTAM | -0.203 | 3.52697E-06 | -0.205 | 2.8812E-06 |
| CSMD3 | 0.260 | 2.15223E-09 | 0.273 | 2.98985E-10 |
| CT62 | 0.229 | 1.57892E-07 | 0.240 | 3.59927E-08 |
| CUL9 | -0.200 | 5.01783E-06 | -0.271 | 4.56876E-10 |
| CX3CL1 | -0.223 | 3.37714E-07 | -0.266 | 8.85422E-10 |
| CXCR6 | -0.216 | 7.83848E-07 | -0.230 | 1.36158E-07 |
| CYP4Z1 | -0.218 | 5.95841E-07 | -0.222 | 3.67458E-07 |
| DCUN1D5 | 0.628 | 1.31456E-57 | 0.454 | 1.9335E-27 |
| DKK3 | 0.208 | 1.91298E-06 | 0.241 | 3.24043E-08 |
| DLX1 | 0.235 | 6.77053E-08 | 0.266 | 9.33423E-10 |
| DLX2 | 0.210 | 1.50793E-06 | 0.312 | 4.83378E-13 |
| DNAJC6 | 0.202 | 4.0328E-06 | 0.280 | 1.01082E-10 |
| DYNC2H1 | 0.367 | 8.05813E-18 | 0.475 | 3.07453E-30 |
| E2F2 | -0.220 | 5.07695E-07 | -0.288 | 2.74721E-11 |
| EFCAB6 | -0.209 | 1.89076E-06 | -0.229 | 1.55856E-07 |
| EFEMP1 | 0.209 | 1.78169E-06 | 0.262 | 1.56708E-09 |
| EHD2 | 0.246 | 1.66177E-08 | 0.326 | 3.33934E-14 |
| EMP2 | -0.244 | 2.15856E-08 | -0.262 | 1.80897E-09 |
| ENDOD1 | 0.255 | 4.96761E-09 | 0.383 | 2.16217E-19 |
| ERO1LB | -0.225 | 2.55731E-07 | -0.269 | 6.00952E-10 |
| EXT2 | 0.226 | 2.24937E-07 | 0.298 | 5.52253E-12 |
| EYA2 | -0.212 | 1.27958E-06 | -0.236 | 5.93911E-08 |
| EZH2 | -0.234 | 7.92497E-08 | -0.270 | 5.42062E-10 |
| F3 | 0.216 | 7.9454E-07 | 0.336 | 4.83092E-15 |
| FAM149A | -0.223 | 3.29049E-07 | -0.257 | 3.50547E-09 |
| FAM3B | -0.219 | 5.49434E-07 | -0.221 | 4.30153E-07 |
| FAM53B | -0.212 | 1.29025E-06 | -0.200 | 5.15087E-06 |
| FAM83E | -0.214 | 1.01528E-06 | -0.253 | 6.37659E-09 |
| FASLG | -0.206 | 2.51903E-06 | -0.239 | 4.30291E-08 |
| FBF1 | -0.259 | 2.46453E-09 | -0.278 | 1.3625E-10 |
| FBLIM1 | 0.211 | 1.38241E-06 | 0.291 | 1.67335E-11 |
| FCHSD2 | 0.250 | 9.28904E-09 | 0.343 | 1.4014E-15 |
| FEZ1 | 0.247 | 1.53563E-08 | 0.326 | 3.86246E-14 |
| FGF5 | 0.250 | 9.33835E-09 | 0.311 | 5.25869E-13 |
| FLII | 0.205 | 2.68962E-06 | 0.259 | 2.66467E-09 |
| FLJ44054 | 0.252 | 6.8272E-09 | 0.220 | 4.58401E-07 |
| FOLR3 | 0.317 | 1.81832E-13 | 0.293 | 1.38746E-11 |
| FRMD5 | 0.246 | 1.6313E-08 | 0.241 | 3.33497E-08 |
| FSCN1 | 0.210 | 1.55371E-06 | 0.286 | 3.98239E-11 |
| FSTL3 | 0.316 | 2.25829E-13 | 0.222 | 3.74615E-07 |
| GABRP | -0.231 | 1.1708E-07 | -0.203 | 3.43752E-06 |
| GAS6 | 0.233 | 9.44471E-08 | 0.200 | 5.16353E-06 |
| GLP2R | 0.266 | 8.9632E-10 | 0.280 | 1.08422E-10 |
| GRIN2C | -0.200 | 4.99941E-06 | -0.301 | 3.55155E-12 |
| GRIPAP1 | -0.220 | 4.85682E-07 | -0.312 | 4.76532E-13 |
| GZMH | -0.207 | 2.24478E-06 | -0.332 | 1.13016E-14 |
| GZMM | -0.212 | 1.33078E-06 | -0.331 | 1.34333E-14 |
| HACL1 | -0.211 | 1.36225E-06 | -0.306 | 1.47275E-12 |
| HTR1D | 0.230 | 1.43245E-07 | 0.227 | 1.94978E-07 |
| ICA1 | -0.230 | 1.41077E-07 | -0.305 | 1.71455E-12 |
| IGFBP6 | 0.236 | 6.72428E-08 | 0.220 | 5.02279E-07 |
| IL17RB | -0.216 | 8.11308E-07 | -0.252 | 7.43962E-09 |
| INTS4 | 0.238 | 5.03212E-08 | 0.269 | 5.57921E-10 |
| IQCG | -0.220 | 4.63089E-07 | -0.208 | 2.12042E-06 |
| IRX6 | -0.263 | 1.52756E-09 | -0.320 | 1.05546E-13 |
| ITGA5 | 0.200 | 5.0059E-06 | 0.229 | 1.55149E-07 |
| JPH2 | 0.239 | 3.98428E-08 | 0.263 | 1.38221E-09 |
| JRKL | 0.243 | 2.49888E-08 | 0.489 | 3.40696E-32 |
| KANK4 | 0.238 | 4.95467E-08 | 0.273 | 3.06129E-10 |
| KCTD21 | 0.252 | 6.83577E-09 | 0.431 | 1.13775E-24 |
| KHDC1L | 0.213 | 1.09261E-06 | 0.206 | 2.4371E-06 |
| KIAA1377 | 0.447 | 1.25132E-26 | 0.392 | 2.41518E-20 |
| KIAA1407 | -0.232 | 1.03876E-07 | -0.225 | 2.64256E-07 |
| KIAA1609 | 0.220 | 5.02269E-07 | 0.298 | 5.28613E-12 |
| KIF25 | -0.201 | 4.3643E-06 | -0.259 | 2.7044E-09 |
| KIFC3 | 0.248 | 1.31078E-08 | 0.209 | 1.85753E-06 |
| KLK5 | 0.206 | 2.61071E-06 | 0.305 | 1.58982E-12 |
| KLRB1 | -0.241 | 3.38126E-08 | -0.292 | 1.62366E-11 |
| KLRG1 | -0.230 | 1.40646E-07 | -0.261 | 2.0913E-09 |
| KLRK1 | -0.219 | 5.27524E-07 | -0.307 | 1.07918E-12 |
| KRT17 | 0.202 | 3.86932E-06 | 0.256 | 3.9176E-09 |
| KRTAP2-1 | 0.319 | 1.33021E-13 | 0.218 | 5.83766E-07 |
| L1CAM | 0.272 | 3.55805E-10 | 0.310 | 6.77972E-13 |
| LETM2 | 0.328 | 2.4264E-14 | 0.286 | 4.19203E-11 |
| LHX2 | -0.218 | 6.09481E-07 | -0.244 | 2.12922E-08 |
| LIG1 | -0.208 | 2.0351E-06 | -0.345 | 7.84403E-16 |
| LMO4 | -0.233 | 9.19391E-08 | -0.260 | 2.16592E-09 |
| LOC100132832 | -0.218 | 5.98508E-07 | -0.202 | 3.838E-06 |
| LOC100188949 | -0.215 | 9.17891E-07 | -0.283 | 6.23703E-11 |
| LOC100216001 | 0.220 | 4.76167E-07 | 0.204 | 3.30198E-06 |
| LOC340508 | 0.230 | 1.33422E-07 | 0.230 | 1.33847E-07 |
| LOC653566 | 0.286 | 4.37028E-11 | 0.232 | 1.03416E-07 |
| LOC727896 | 0.201 | 4.58451E-06 | 0.461 | 2.5865E-28 |
| LRRN4 | 0.316 | 2.41179E-13 | 0.281 | 8.50563E-11 |
| MAP3K14 | -0.264 | 1.28769E-09 | -0.296 | 7.75071E-12 |
| MAP4K1 | -0.210 | 1.67801E-06 | -0.324 | 4.92823E-14 |
| MAP7D3 | 0.208 | 1.97517E-06 | 0.249 | 1.13293E-08 |
| MCCC1 | -0.231 | 1.17668E-07 | -0.247 | 1.49672E-08 |
| MCM5 | -0.202 | 3.839E-06 | -0.362 | 2.56038E-17 |
| MDH1 | -0.207 | 2.26316E-06 | -0.289 | 2.58036E-11 |
| MED17 | 0.314 | 3.09147E-13 | 0.520 | 7.48816E-37 |
| MEI1 | -0.245 | 2.00325E-08 | -0.347 | 6.17364E-16 |
| METTL7A | -0.237 | 5.6392E-08 | -0.228 | 1.85526E-07 |
| MGAT3 | -0.251 | 8.55114E-09 | -0.203 | 3.37401E-06 |
| MMP1 | 0.202 | 3.90968E-06 | 0.232 | 1.0052E-07 |
| MMP10 | 0.260 | 2.27225E-09 | 0.314 | 3.09663E-13 |
| MMP13 | 0.207 | 2.35167E-06 | 0.228 | 1.74071E-07 |
| MSX2 | 0.206 | 2.66702E-06 | 0.258 | 2.97383E-09 |
| MTMR2 | 0.327 | 3.22787E-14 | 0.528 | 3.39149E-38 |
| MYB | -0.218 | 6.19461E-07 | -0.252 | 7.40335E-09 |
| MYO3A | -0.225 | 2.65919E-07 | -0.249 | 1.14794E-08 |
| NARS2 | 0.244 | 2.14463E-08 | 0.341 | 2.10865E-15 |
| NEFH | -0.237 | 5.7393E-08 | -0.276 | 2.02368E-10 |
| NGF | 0.223 | 3.43981E-07 | 0.200 | 4.88087E-06 |
| NLRC3 | -0.204 | 3.14201E-06 | -0.211 | 1.41409E-06 |
| NT5E | 0.240 | 3.69816E-08 | 0.321 | 9.53638E-14 |
| NUMA1 | 0.210 | 1.5616E-06 | 0.288 | 2.87328E-11 |
| NUP210 | -0.241 | 3.16424E-08 | -0.259 | 2.51483E-09 |
| NXN | 0.210 | 1.64001E-06 | 0.212 | 1.3279E-06 |
| OLFM1 | -0.215 | 9.26873E-07 | -0.237 | 5.29601E-08 |
| OSTalpha | -0.244 | 2.2653E-08 | -0.302 | 2.62942E-12 |
| OXER1 | -0.220 | 4.98589E-07 | -0.373 | 2.22238E-18 |
| P2RY2 | 0.207 | 2.20209E-06 | 0.329 | 1.94842E-14 |
| PAAF1 | 0.329 | 1.87523E-14 | 0.203 | 3.52285E-06 |
| PANX1 | 0.318 | 1.63824E-13 | 0.514 | 5.4228E-36 |
| PARVA | 0.204 | 3.27938E-06 | 0.303 | 2.37808E-12 |
| PEA15 | 0.210 | 1.52236E-06 | 0.249 | 1.06867E-08 |
| PICALM | 0.304 | 1.86829E-12 | 0.541 | 2.55217E-40 |
| PIK3R3 | -0.283 | 6.33849E-11 | -0.248 | 1.18316E-08 |
| PLA2G2D | -0.203 | 3.39273E-06 | -0.241 | 3.22833E-08 |
| PLCB3 | 0.244 | 2.06521E-08 | 0.203 | 3.5365E-06 |
| PLCD3 | 0.214 | 9.77193E-07 | 0.304 | 1.89723E-12 |
| POU2AF1 | -0.203 | 3.68345E-06 | -0.202 | 3.91694E-06 |
| PPME1 | 0.294 | 1.07632E-11 | 0.301 | 3.542E-12 |
| PPP1R3E | -0.200 | 4.91864E-06 | -0.231 | 1.19827E-07 |
| PPP1R3F | -0.203 | 3.68207E-06 | -0.296 | 8.18296E-12 |
| PPT2 | 0.209 | 1.90178E-06 | 0.218 | 5.86132E-07 |
| PRDM15 | -0.256 | 4.26529E-09 | -0.289 | 2.54753E-11 |
| PRKAB2 | 0.208 | 2.03522E-06 | 0.246 | 1.67617E-08 |
| PRSS23 | 0.295 | 8.68805E-12 | 0.325 | 4.07723E-14 |
| PSG1 | 0.298 | 5.6699E-12 | 0.279 | 1.16638E-10 |
| PSG11 | 0.270 | 4.91658E-10 | 0.260 | 2.32558E-09 |
| PSG2 | 0.274 | 2.62095E-10 | 0.298 | 5.34099E-12 |
| PSG3 | 0.261 | 1.97986E-09 | 0.219 | 5.59968E-07 |
| PSG4 | 0.219 | 5.34863E-07 | 0.282 | 7.58504E-11 |
| PSG5 | 0.225 | 2.60807E-07 | 0.233 | 9.64721E-08 |
| PSG6 | 0.262 | 1.72096E-09 | 0.252 | 7.08859E-09 |
| PSG7 | 0.241 | 3.09333E-08 | 0.235 | 7.30833E-08 |
| PSPC1 | -0.220 | 4.89648E-07 | -0.213 | 1.07344E-06 |
| PTRF | 0.245 | 1.8979E-08 | 0.295 | 9.19214E-12 |
| PVRIG | -0.219 | 5.50982E-07 | -0.306 | 1.48215E-12 |
| PXN | 0.233 | 9.57915E-08 | 0.293 | 1.19448E-11 |
| PYGL | 0.230 | 1.32991E-07 | 0.262 | 1.77086E-09 |
| QSOX1 | 0.227 | 1.97248E-07 | 0.296 | 7.97843E-12 |
| RAB11FIP5 | 0.236 | 6.16163E-08 | 0.322 | 8.30822E-14 |
| RAB38 | 0.269 | 5.94944E-10 | 0.364 | 1.69102E-17 |
| RAB6A | 0.267 | 8.17586E-10 | 0.481 | 4.92497E-31 |
| RALGPS1 | -0.279 | 1.22688E-10 | -0.231 | 1.24557E-07 |
| RANBP17 | -0.234 | 7.77042E-08 | -0.252 | 7.17782E-09 |
| RAPGEF3 | 0.294 | 1.16375E-11 | 0.309 | 7.7951E-13 |
| RELT | 0.293 | 1.26701E-11 | 0.255 | 4.91154E-09 |
| RFPL1S | -0.219 | 5.41697E-07 | -0.273 | 3.23057E-10 |
| RHBDL3 | -0.245 | 1.82662E-08 | -0.249 | 1.0516E-08 |
| RHOD | 0.254 | 5.38686E-09 | 0.215 | 9.014E-07 |
| RPS6KA4 | 0.225 | 2.48888E-07 | 0.216 | 8.00425E-07 |
| RRAS2 | 0.293 | 1.30105E-11 | 0.350 | 3.24504E-16 |
| SAMD4A | 0.215 | 9.26795E-07 | 0.320 | 1.03412E-13 |
| SAP30L | -0.200 | 4.90881E-06 | -0.208 | 2.09187E-06 |
| SCML4 | -0.218 | 6.23541E-07 | -0.260 | 2.20845E-09 |
| SEMA4D | -0.265 | 1.12291E-09 | -0.253 | 6.38961E-09 |
| SERPINH1 | 0.272 | 3.64809E-10 | 0.251 | 8.09113E-09 |
| SFI1 | -0.215 | 8.6615E-07 | -0.400 | 4.28036E-21 |
| SFRS2B | 0.217 | 7.05702E-07 | 0.264 | 1.3466E-09 |
| SFTA1P | 0.409 | 4.15465E-22 | 0.351 | 2.7492E-16 |
| SFXN3 | 0.224 | 2.92135E-07 | 0.237 | 5.2914E-08 |
| SH2D1A | -0.215 | 8.73396E-07 | -0.241 | 3.10274E-08 |
| SH2D5 | 0.221 | 4.02327E-07 | 0.282 | 8.29206E-11 |
| SH3TC2 | 0.226 | 2.25724E-07 | 0.372 | 2.51909E-18 |
| SIGLEC8 | -0.203 | 3.55893E-06 | -0.222 | 3.64093E-07 |
| SKAP1 | -0.243 | 2.45795E-08 | -0.393 | 1.94926E-20 |
| SLC12A4 | 0.232 | 1.06505E-07 | 0.295 | 9.45012E-12 |
| SLC16A2 | 0.241 | 3.31981E-08 | 0.293 | 1.18604E-11 |
| SLC22A15 | 0.222 | 3.92704E-07 | 0.282 | 7.78996E-11 |
| SLC36A4 | 0.216 | 7.67059E-07 | 0.225 | 2.71555E-07 |
| SLC38A4 | 0.248 | 1.25459E-08 | 0.394 | 1.78039E-20 |
| SLC38A5 | 0.228 | 1.88216E-07 | 0.215 | 8.46516E-07 |
| SLC6A2 | 0.265 | 1.1198E-09 | 0.303 | 2.41122E-12 |
| SLC7A5 | 0.251 | 8.09297E-09 | 0.329 | 1.86209E-14 |
| SNORA8 | 0.307 | 1.15314E-12 | 0.311 | 5.44387E-13 |
| SNX33 | 0.230 | 1.33521E-07 | 0.323 | 6.46149E-14 |
| SPANXB2 | 0.234 | 7.89014E-08 | 0.214 | 1.00131E-06 |
| SPRYD3 | 0.221 | 4.38453E-07 | 0.216 | 8.29332E-07 |
| SRPX2 | 0.216 | 8.15118E-07 | 0.232 | 1.08356E-07 |
| STAG3 | -0.205 | 2.78822E-06 | -0.272 | 3.80847E-10 |
| STX3 | 0.203 | 3.52826E-06 | 0.350 | 2.82972E-16 |
| SVIP | -0.208 | 1.90541E-06 | -0.234 | 7.8852E-08 |
| SYCP2 | -0.225 | 2.61322E-07 | -0.276 | 2.05749E-10 |
| SYT16 | 0.272 | 3.48187E-10 | 0.293 | 1.39926E-11 |
| TAF1D | 0.411 | 2.57319E-22 | 0.289 | 2.71056E-11 |
| TBX21 | -0.210 | 1.65908E-06 | -0.281 | 8.83721E-11 |
| THSD1 | 0.207 | 2.34148E-06 | 0.300 | 4.09002E-12 |
| TIGIT | -0.207 | 2.18638E-06 | -0.211 | 1.37306E-06 |
| TLX3 | -0.228 | 1.88332E-07 | -0.296 | 8.05542E-12 |
| TMC8 | -0.228 | 1.7374E-07 | -0.324 | 4.83207E-14 |
| TMEM123 | 0.699 | 1.72979E-76 | 0.744 | 1.60908E-91 |
| TMEM133 | 0.321 | 9.2581E-14 | 0.397 | 8.12295E-21 |
| TMEM135 | 0.209 | 1.86526E-06 | 0.272 | 3.92141E-10 |
| TNFRSF12A | 0.233 | 9.56391E-08 | 0.233 | 9.74602E-08 |
| TSKU | 0.233 | 9.42736E-08 | 0.258 | 2.82675E-09 |
| TSPAN10 | 0.250 | 9.56404E-09 | 0.231 | 1.26843E-07 |
| TUBB6 | 0.218 | 6.2124E-07 | 0.212 | 1.20283E-06 |
| UBASH3A | -0.249 | 1.13351E-08 | -0.272 | 3.96045E-10 |
| UBD | -0.258 | 3.16746E-09 | -0.320 | 1.03358E-13 |
| UCA1 | 0.215 | 9.18995E-07 | 0.280 | 1.15628E-10 |
| VAT1 | 0.229 | 1.65792E-07 | 0.248 | 1.27462E-08 |
| VEGFC | 0.219 | 5.597E-07 | 0.241 | 3.15499E-08 |
| VGLL1 | 0.202 | 4.1799E-06 | 0.269 | 5.82427E-10 |
| VSIG1 | 0.202 | 3.9843E-06 | 0.328 | 2.4487E-14 |
| WNK2 | -0.214 | 1.04997E-06 | -0.240 | 3.60691E-08 |
| WNT7A | 0.216 | 8.1393E-07 | 0.263 | 1.43371E-09 |
| YAP1 | 0.781 | 1.7773E-106 | 1.000 | 0 |
| ZFR2 | -0.216 | 8.26062E-07 | -0.334 | 7.45564E-15 |
| ZMAT1 | -0.266 | 9.84239E-10 | -0.272 | 3.83425E-10 |
| ZNF683 | -0.205 | 2.85851E-06 | -0.303 | 2.15718E-12 |
