# Supplementary material for: Clinical significance of YAP1 activation in head and neck squamous cell carcinoma
Source: Oncotarget. 2017 Nov 27;8(67):111130–43. doi: 10.18632/oncotarget.22666 (PMC5762311; doi:10.18632/oncotarget.22666)
Supplement: Supplementary file 3 [file oncotarget-08-111130-s003.docx]

| **Supplementary Table 2. Fisher’s exact test p values for frequency comparisons of significantly reoccurring alterations by YAP1 subtypes.** | | | | | |
| --- | --- | --- | --- | --- | --- |
|  |  |  | | | |
| **Amplifications** | | | **Deletion** | | |
| Peak | Potential driver | YA VS YI | Peak | Potential driver | YA VS YI |
| 01q21.2 |  | 0.002969 | 2q21.2 |  |  |
| 02q11.2 |  | 0.5398 | 2q22.1 |  |  |
| 03q26.32 | PIK3CA | 0.0001113 | 1p36.13 |  | 0.001935 |
| 03q26.33 | SOX2 | 0.00004336 | 1p13.2 |  | 1 |
| 03q28 | tp63 | 0.00003515 | 2q21.2 |  | 0.5656 |
| 5p12 |  |  | 2q22.1 | LRP1B | 0.7628 |
| 5p15.33 |  | 0.8204 | 2q36.2 |  | 0.3179 |
| 07p11.2 | EGFR | 0.001943 | 3p14.3 |  | 0.01026 |
| 8p11.23 | FGFR1 | 0.9019 | 3p14.2 | FHIT | 0.005536 |
| 8q11.21 | SNAI2? | 0.0453 | 3p14.2 |  | 0.01977 |
| 8q24.21 | MYC | 0.9622 | 3p13 |  | 0.02608 |
| 11q13.3 | FADD | 0.08256 | 4p16.2 |  | 0.09227 |
| 13q22.1 | KLF1 |  | 4q22.1 |  | 1 |
| 14q11.2 |  | 0.05169 | 4q31.3 |  | 1 |
| 17q12 | ERBB2 | 0.06388 | 4q35.2 | FAT1 | 0.3967 |
| 20q11 | src | 0.7094 | 5q11.2 |  | 0.04931 |
| 20q11.22 | E2F1 | 0.08517 | 5q15 |  | 0.041 |
|  |  |  | 5q35.3 |  | 0.07343 |
|  |  |  | 6p25.3 |  | 0.405 |
|  |  |  | 6q12 |  | 0.06684 |
|  |  |  | 7q36.1 |  | 0.8334 |
|  |  |  | 8p23.2 | CSMD1 | 5.003E-09 |
|  |  |  | 8p23.3 |  | 4.054E-07 |
|  |  |  | 9p23 | PTPRD | 0.003305 |
|  |  |  | 9p21.3 | CDKN2A | 0.0001693 |
|  |  |  | 9q21.11 |  | 0.06199 |
|  |  |  | 9q34.3 | NOTCH1 | 0.004153 |
|  |  |  | 10p11.21 |  | 0.0004903 |
|  |  |  | 10q23.31 | PTEN | 0.4711 |
|  |  |  | 11p15.4 |  | 0.00126 |
|  |  |  | 11q23.1 |  | 6.256E-07 |
|  |  |  | 13q12.11 |  | 0.3119 |
|  |  |  | 13q21.33 |  | 0.01176 |
|  |  |  | 14q11.2 |  | 0.6956 |
|  |  |  | 14q32.32 |  | 0.000006187 |
|  |  |  | 15q15.1 |  | 0.1981 |
|  |  |  | 16q12.1 |  | 2.026E-07 |
|  |  |  | 16q23.3 |  | 0.0001179 |
|  |  |  | 17q25.3 |  | 0.1084 |
|  |  |  | 18q21.2 | smad4 | 8.535E-08 |
|  |  |  | 18q23 |  | 0.000000338 |
|  |  |  | 19p13.3 |  | 0.008311 |
|  |  |  | 19q13.43 |  | 0.04031 |
|  |  |  | Xp21.3 |  | 0.9208 |
|  |  |  | Xp11.3 | KDM6A | 0.5506 |
