# Supplementary material for: Clinical significance of YAP1 activation in head and neck squamous cell carcinoma
Source: Oncotarget. 2017 Nov 27;8(67):111130–43. doi: 10.18632/oncotarget.22666 (PMC5762311; doi:10.18632/oncotarget.22666)
Supplement: Supplementary file 4 [file oncotarget-08-111130-s004.docx]

| **Supplementary Table 3. comparison of somatic mutation frequencies between YA and YI subtypes.** | | | |
| --- | --- | --- | --- |
|  |  |  | |
|  | YA | YI | Fisher's p value |
| TP53_MUT | 152 | 199 | 1.40E-05 |
| TP53_WT | 32 | 110 |  |
| NFE2L2_MUT | 2 | 24 | 0.0006959 |
| NFE2L2_WT | 182 | 285 |  |
| CDKN2A_MUT | 52 | 59 | 0.01965 |
| CDKN2A_WT | 132 | 250 |  |
| PTEN_MUT | 1 | 13 | 0.02176 |
| PTEN_WT | 183 | 296 |  |
| SYNE1_MUT | 26 | 69 | 0.02549 |
| SYNE1_WT | 158 | 240 |  |
| NSD1_MUT | 15 | 46 | 0.0334 |
| NSD1_WT | 169 | 263 |  |
| DICER1_MUT | 1 | 10 | 0.06021 |
| DICER1_WT | 183 | 299 |  |
| IRF6_MUT | 0 | 6 | 0.08868 |
| IRF6_WT | 184 | 303 |  |
| CUL3_MUT | 2 | 12 | 0.09275 |
| CUL3_WT | 182 | 297 |  |
| PIK3CA_MUT | 27 | 63 | 0.1187 |
| PIK3CA_WT | 157 | 246 |  |
| NOTCH1_MUT | 38 | 47 | 0.1392 |
| NOTCH1_WT | 146 | 262 |  |
| HRAS_MUT | 14 | 13 | 0.1505 |
| HRAS_WT | 170 | 296 |  |
| PIK3R1_MUT | 1 | 8 | 0.1635 |
| PIK3R1_WT | 183 | 301 |  |
| RIPK4_MUT | 1 | 8 | 0.1635 |
| RIPK4_WT | 183 | 301 |  |
| FBXW7_MUT | 8 | 24 | 0.1851 |
| FBXW7_WT | 176 | 285 |  |
| RB1_MUT | 4 | 14 | 0.2196 |
| RB1_WT | 180 | 295 |  |
| TP63_MUT | 2 | 10 | 0.2254 |
| TP63_WT | 182 | 299 |  |
| HLA-A_MUT | 14 | 15 | 0.2368 |
| HLA-A_WT | 170 | 294 |  |
| TRAF3_MUT | 0 | 4 | 0.302 |
| TRAF3_WT | 184 | 305 |  |
| TGFBR2_MUT | 11 | 12 | 0.3772 |
| TGFBR2_WT | 173 | 297 |  |
| CASP8_MUT | 23 | 31 | 0.4563 |
| CASP8_WT | 161 | 278 |  |
| NOTCH2_MUT | 10 | 13 | 0.5177 |
| NOTCH2_WT | 174 | 296 |  |
| AJUBA_MUT | 13 | 18 | 0.5716 |
| AJUBA_WT | 171 | 291 |  |
| NOTCH3_MUT | 7 | 16 | 0.6595 |
| NOTCH3_WT | 177 | 293 |  |
| SYNE2_MUT | 15 | 22 | 0.7248 |
| SYNE2_WT | 169 | 287 |  |
| MED1_MUT | 3 | 7 | 0.7504 |
| MED1_WT | 181 | 302 |  |
| KMT2D_MUT | 28 | 51 | 0.7998 |
| KMT2D_WT | 156 | 258 |  |
| FAT1_MUT | 41 | 73 | 0.8253 |
| FAT1_WT | 143 | 236 |  |
| MLL2_MUT | 0 | 0 | 1 |
| MLL2_WT | 184 | 309 |  |
| EZH2_MUT | 2 | 3 | 1 |
| EZH2_WT | 182 | 306 |  |
