# Supplementary material for: Clinical significance of YAP1 activation in head and neck squamous cell carcinoma
Source: Oncotarget. 2017 Nov 27;8(67):111130–43. doi: 10.18632/oncotarget.22666 (PMC5762311; doi:10.18632/oncotarget.22666)
Supplement: Supplementary file 5 [file oncotarget-08-111130-s005.docx]

| **Supplementary Table 7. Molecular classification and YAP1 subtype** | | |
| --- | --- | --- |
| Sample id | Molecular classification | YAP1 subtype |
| TCGA-4P-AA8J | mesenchymal | YA |
| TCGA-BA-4074 | classical | YA |
| TCGA-BA-4075 | basal | YA |
| TCGA-BA-4076 | basal | YI |
| TCGA-BA-4077 | atypical | YI |
| TCGA-BA-4078 | classical | YI |
| TCGA-BA-5149 | basal | YA |
| TCGA-BA-5151 | basal | YA |
| TCGA-BA-5152 | basal | YI |
| TCGA-BA-5153 | atypical | YI |
| TCGA-BA-5555 | classical | YI |
| TCGA-BA-5556 | basal | YI |
| TCGA-BA-5557 | basal | YA |
| TCGA-BA-5558 | basal | YA |
| TCGA-BA-5559 | mesenchymal | YI |
| TCGA-BA-6868 | classical | YA |
| TCGA-BA-6869 | atypical | YI |
| TCGA-BA-6870 | basal | YA |
| TCGA-BA-6871 | mesenchymal | YI |
| TCGA-BA-6872 | basal | YA |
| TCGA-BA-6873 | basal | YA |
| TCGA-BA-7269 | mesenchymal | YI |
| TCGA-BA-A4IF | classical | YI |
| TCGA-BA-A4IG | atypical | YI |
| TCGA-BA-A4IH | atypical | YI |
| TCGA-BA-A4II | basal | YI |
| TCGA-BA-A6D8 | classical | YI |
| TCGA-BA-A6DA | basal | YI |
| TCGA-BA-A6DB | atypical | YI |
| TCGA-BA-A6DD | atypical | YI |
| TCGA-BA-A6DE | basal | YA |
| TCGA-BA-A6DG | mesenchymal | YA |
| TCGA-BA-A6DI | atypical | YA |
| TCGA-BA-A6DJ | basal | YA |
| TCGA-BA-A6DL | atypical | YI |
| TCGA-BA-A8YP | classical | YI |
| TCGA-BB-4217 | atypical | YI |
| TCGA-BB-4223 | atypical | YI |
| TCGA-BB-4224 | basal | YI |
| TCGA-BB-4225 | atypical | YI |
| TCGA-BB-4227 | atypical | YA |
| TCGA-BB-4228 | atypical | YI |
| TCGA-BB-7861 | atypical | YI |
| TCGA-BB-7862 | classical | YI |
| TCGA-BB-7863 | basal | YA |
| TCGA-BB-7864 | classical | YI |
| TCGA-BB-7866 | atypical | YI |
| TCGA-BB-7870 | classical | YI |
| TCGA-BB-7871 | classical | YI |
| TCGA-BB-7872 | basal | YA |
| TCGA-BB-8596 | mesenchymal | YI |
| TCGA-BB-8601 | classical | YI |
| TCGA-BB-A5HU | mesenchymal | YA |
| TCGA-BB-A5HY | classical | YA |
| TCGA-BB-A5HZ | basal | YA |
| TCGA-BB-A6UM | atypical | YI |
| TCGA-BB-A6UO | basal | YA |
| TCGA-C9-A47Z | basal | YA |
| TCGA-C9-A480 | basal | YA |
| TCGA-CN-4722 | basal | YI |
| TCGA-CN-4723 | classical | YI |
| TCGA-CN-4725 | basal | YA |
| TCGA-CN-4726 | basal | YA |
| TCGA-CN-4727 | classical | YA |
| TCGA-CN-4728 | mesenchymal | YI |
| TCGA-CN-4729 | basal | YA |
| TCGA-CN-4730 | basal | YA |
| TCGA-CN-4731 | atypical | YA |
| TCGA-CN-4733 | mesenchymal | YI |
| TCGA-CN-4735 | classical | YI |
| TCGA-CN-4736 | mesenchymal | YI |
| TCGA-CN-4737 | mesenchymal | YA |
| TCGA-CN-4738 | atypical | YI |
| TCGA-CN-4739 | atypical | YI |
| TCGA-CN-4740 | mesenchymal | YI |
| TCGA-CN-4741 | atypical | YI |
| TCGA-CN-4742 | basal | YA |
| TCGA-CN-5355 | mesenchymal | YA |
| TCGA-CN-5356 | mesenchymal | YI |
| TCGA-CN-5358 | mesenchymal | YI |
| TCGA-CN-5359 | mesenchymal | YI |
| TCGA-CN-5360 | atypical | YI |
| TCGA-CN-5363 | mesenchymal | YA |
| TCGA-CN-5364 | atypical | YI |
| TCGA-CN-5365 | basal | YI |
| TCGA-CN-5366 | basal | YI |
| TCGA-CN-5369 | mesenchymal | YI |
| TCGA-CN-5370 | mesenchymal | YA |
| TCGA-CN-5373 | basal | YA |
| TCGA-CN-5374 | atypical | YI |
| TCGA-CN-6010 | classical | YI |
| TCGA-CN-6011 | basal | YA |
| TCGA-CN-6012 | classical | YI |
| TCGA-CN-6016 | mesenchymal | YI |
| TCGA-CN-6017 | mesenchymal | YI |
| TCGA-CN-6018 | mesenchymal | YA |
| TCGA-CN-6019 | mesenchymal | YI |
| TCGA-CN-6020 | basal | YA |
| TCGA-CN-6021 | classical | YI |
| TCGA-CN-6022 | mesenchymal | YA |
| TCGA-CN-6023 | classical | YI |
| TCGA-CN-6024 | classical | YI |
| TCGA-CN-6988 | atypical | YI |
| TCGA-CN-6989 | mesenchymal | YI |
| TCGA-CN-6992 | classical | YI |
| TCGA-CN-6994 | mesenchymal | YI |
| TCGA-CN-6995 | basal | YI |
| TCGA-CN-6996 | basal | YA |
| TCGA-CN-6997 | classical | YI |
| TCGA-CN-6998 | basal | YA |
| TCGA-CN-A498 | basal | YI |
| TCGA-CN-A49A | classical | YI |
| TCGA-CN-A49C | atypical | YI |
| TCGA-CN-A63U | classical | YI |
| TCGA-CN-A63W | classical | YA |
| TCGA-CN-A641 | classical | YI |
| TCGA-CN-A6UY | atypical | YI |
| TCGA-CN-A6V1 | atypical | YI |
| TCGA-CN-A6V3 | atypical | YI |
| TCGA-CN-A6V6 | atypical | YI |
| TCGA-CN-A6V7 | atypical | YI |
| TCGA-CQ-5323 | atypical | YI |
| TCGA-CQ-5324 | basal | YI |
| TCGA-CQ-5325 | mesenchymal | YI |
| TCGA-CQ-5326 | basal | YA |
| TCGA-CQ-5327 | basal | YI |
| TCGA-CQ-5329 | mesenchymal | YA |
| TCGA-CQ-5330 | mesenchymal | YA |
| TCGA-CQ-5331 | basal | YI |
| TCGA-CQ-5332 | classical | YI |
| TCGA-CQ-5333 | mesenchymal | YI |
| TCGA-CQ-5334 | mesenchymal | YA |
| TCGA-CQ-6218 | basal | YA |
| TCGA-CQ-6220 | basal | YA |
| TCGA-CQ-6221 | mesenchymal | YI |
| TCGA-CQ-6223 | basal | YI |
| TCGA-CQ-6224 | mesenchymal | YA |
| TCGA-CQ-6225 | classical | YI |
| TCGA-CQ-6227 | mesenchymal | YA |
| TCGA-CQ-6228 | classical | YI |
| TCGA-CQ-6229 | basal | YI |
| TCGA-CQ-7063 | basal | YA |
| TCGA-CQ-7065 | basal | YA |
| TCGA-CQ-7068 | basal | YI |
| TCGA-CQ-7069 | basal | YA |
| TCGA-CQ-7071 | basal | YA |
| TCGA-CQ-7072 | mesenchymal | YA |
| TCGA-CQ-A4C6 | basal | YA |
| TCGA-CQ-A4C7 | mesenchymal | YA |
| TCGA-CQ-A4C9 | atypical | YI |
| TCGA-CQ-A4CA | mesenchymal | YI |
| TCGA-CQ-A4CB | mesenchymal | YA |
| TCGA-CQ-A4CD | basal | YA |
| TCGA-CQ-A4CE | basal | YI |
| TCGA-CQ-A4CG | classical | YA |
| TCGA-CQ-A4CH | basal | YA |
| TCGA-CQ-A4CI | classical | YI |
| TCGA-CR-5243 | atypical | YI |
| TCGA-CR-5247 | classical | YA |
| TCGA-CR-5248 | atypical | YI |
| TCGA-CR-5249 | atypical | YI |
| TCGA-CR-5250 | atypical | YI |
| TCGA-CR-6467 | atypical | YI |
| TCGA-CR-6470 | atypical | YI |
| TCGA-CR-6471 | basal | YI |
| TCGA-CR-6472 | atypical | YI |
| TCGA-CR-6474 | mesenchymal | YA |
| TCGA-CR-6477 | mesenchymal | YI |
| TCGA-CR-6478 | mesenchymal | YI |
| TCGA-CR-6480 | atypical | YI |
| TCGA-CR-6481 | atypical | YI |
| TCGA-CR-6482 | atypical | YI |
| TCGA-CR-6484 | mesenchymal | YI |
| TCGA-CR-6487 | atypical | YI |
| TCGA-CR-6488 | mesenchymal | YA |
| TCGA-CR-6491 | basal | YA |
| TCGA-CR-6492 | atypical | YI |
| TCGA-CR-6493 | basal | YA |
| TCGA-CR-7364 | atypical | YI |
| TCGA-CR-7365 | basal | YA |
| TCGA-CR-7367 | mesenchymal | YA |
| TCGA-CR-7368 | atypical | YI |
| TCGA-CR-7369 | atypical | YI |
| TCGA-CR-7370 | classical | YI |
| TCGA-CR-7371 | classical | YI |
| TCGA-CR-7372 | basal | YA |
| TCGA-CR-7373 | basal | YA |
| TCGA-CR-7374 | classical | YI |
| TCGA-CR-7376 | mesenchymal | YI |
| TCGA-CR-7377 | mesenchymal | YI |
| TCGA-CR-7379 | basal | YA |
| TCGA-CR-7380 | basal | YI |
| TCGA-CR-7382 | mesenchymal | YA |
| TCGA-CR-7383 | mesenchymal | YI |
| TCGA-CR-7385 | atypical | YI |
| TCGA-CR-7386 | mesenchymal | YA |
| TCGA-CR-7388 | atypical | YI |
| TCGA-CR-7389 | atypical | YI |
| TCGA-CR-7390 | classical | YA |
| TCGA-CR-7391 | atypical | YI |
| TCGA-CR-7392 | mesenchymal | YA |
| TCGA-CR-7393 | atypical | YI |
| TCGA-CR-7394 | basal | YI |
| TCGA-CR-7395 | basal | YI |
| TCGA-CR-7397 | mesenchymal | YI |
| TCGA-CR-7398 | classical | YI |
| TCGA-CR-7399 | atypical | YI |
| TCGA-CR-7401 | mesenchymal | YA |
| TCGA-CR-7402 | atypical | YI |
| TCGA-CR-7404 | atypical | YI |
| TCGA-CV-5430 | atypical | YI |
| TCGA-CV-5431 | mesenchymal | YI |
| TCGA-CV-5432 | classical | YI |
| TCGA-CV-5434 | classical | YI |
| TCGA-CV-5435 | classical | YA |
| TCGA-CV-5436 | classical | YA |
| TCGA-CV-5439 | basal | YI |
| TCGA-CV-5440 | classical | YI |
| TCGA-CV-5441 | basal | YI |
| TCGA-CV-5442 | basal | YI |
| TCGA-CV-5443 | atypical | YI |
| TCGA-CV-5444 | mesenchymal | YI |
| TCGA-CV-5966 | basal | YA |
| TCGA-CV-5970 | basal | YA |
| TCGA-CV-5971 | mesenchymal | YI |
| TCGA-CV-5973 | basal | YA |
| TCGA-CV-5976 | basal | YA |
| TCGA-CV-5977 | basal | YA |
| TCGA-CV-5978 | classical | YI |
| TCGA-CV-5979 | basal | YA |
| TCGA-CV-6003 | basal | YA |
| TCGA-CV-6433 | atypical | YI |
| TCGA-CV-6436 | basal | YI |
| TCGA-CV-6441 | atypical | YI |
| TCGA-CV-6933 | classical | YI |
| TCGA-CV-6934 | mesenchymal | YI |
| TCGA-CV-6935 | classical | YI |
| TCGA-CV-6937 | mesenchymal | YI |
| TCGA-CV-6938 | basal | YI |
| TCGA-CV-6939 | atypical | YI |
| TCGA-CV-6940 | classical | YI |
| TCGA-CV-6941 | classical | YI |
| TCGA-CV-6942 | mesenchymal | YI |
| TCGA-CV-6943 | mesenchymal | YI |
| TCGA-CV-6945 | classical | YA |
| TCGA-CV-6948 | classical | YI |
| TCGA-CV-6950 | mesenchymal | YA |
| TCGA-CV-6951 | classical | YI |
| TCGA-CV-6952 | basal | YA |
| TCGA-CV-6953 | basal | YI |
| TCGA-CV-6954 | basal | YA |
| TCGA-CV-6955 | atypical | YI |
| TCGA-CV-6956 | basal | YA |
| TCGA-CV-6959 | basal | YA |
| TCGA-CV-6960 | classical | YA |
| TCGA-CV-6961 | mesenchymal | YI |
| TCGA-CV-6962 | classical | YI |
| TCGA-CV-7089 | atypical | YA |
| TCGA-CV-7090 | mesenchymal | YA |
| TCGA-CV-7091 | basal | YA |
| TCGA-CV-7095 | basal | YI |
| TCGA-CV-7097 | mesenchymal | YA |
| TCGA-CV-7099 | basal | YI |
| TCGA-CV-7100 | mesenchymal | YI |
| TCGA-CV-7101 | atypical | YI |
| TCGA-CV-7102 | mesenchymal | YI |
| TCGA-CV-7103 | mesenchymal | YI |
| TCGA-CV-7104 | mesenchymal | YA |
| TCGA-CV-7177 | classical | YI |
| TCGA-CV-7178 | mesenchymal | YI |
| TCGA-CV-7180 | basal | YA |
| TCGA-CV-7183 | basal | YA |
| TCGA-CV-7235 | mesenchymal | YI |
| TCGA-CV-7236 | mesenchymal | YA |
| TCGA-CV-7238 | mesenchymal | YI |
| TCGA-CV-7242 | atypical | YI |
| TCGA-CV-7243 | mesenchymal | YA |
| TCGA-CV-7245 | classical | YA |
| TCGA-CV-7248 | classical | YI |
| TCGA-CV-7250 | classical | YI |
| TCGA-CV-7252 | basal | YA |
| TCGA-CV-7253 | classical | YA |
| TCGA-CV-7254 | mesenchymal | YI |
| TCGA-CV-7255 | basal | YA |
| TCGA-CV-7261 | classical | YI |
| TCGA-CV-7263 | mesenchymal | YI |
| TCGA-CV-7406 | atypical | YI |
| TCGA-CV-7407 | classical | YI |
| TCGA-CV-7410 | mesenchymal | YI |
| TCGA-CV-7411 | basal | YA |
| TCGA-CV-7413 | mesenchymal | YA |
| TCGA-CV-7414 | atypical | YI |
| TCGA-CV-7415 | mesenchymal | YI |
| TCGA-CV-7416 | basal | YA |
| TCGA-CV-7418 | classical | YI |
| TCGA-CV-7421 | mesenchymal | YI |
| TCGA-CV-7422 | classical | YI |
| TCGA-CV-7423 | basal | YA |
| TCGA-CV-7424 | classical | YI |
| TCGA-CV-7425 | basal | YA |
| TCGA-CV-7427 | basal | YI |
| TCGA-CV-7428 | basal | YI |
| TCGA-CV-7429 | classical | YA |
| TCGA-CV-7430 | classical | YI |
| TCGA-CV-7432 | basal | YA |
| TCGA-CV-7433 | mesenchymal | YI |
| TCGA-CV-7434 | basal | YA |
| TCGA-CV-7435 | mesenchymal | YI |
| TCGA-CV-7437 | classical | YI |
| TCGA-CV-7438 | mesenchymal | YA |
| TCGA-CV-7440 | classical | YA |
| TCGA-CV-A45O | basal | YI |
| TCGA-CV-A45P | basal | YA |
| TCGA-CV-A45Q | basal | YI |
| TCGA-CV-A45R | basal | YI |
| TCGA-CV-A45T | basal | YI |
| TCGA-CV-A45U | classical | YI |
| TCGA-CV-A45V | atypical | YI |
| TCGA-CV-A45W | classical | YI |
| TCGA-CV-A45X | basal | YA |
| TCGA-CV-A45Y | mesenchymal | YI |
| TCGA-CV-A45Z | classical | YI |
| TCGA-CV-A460 | atypical | YI |
| TCGA-CV-A461 | atypical | YI |
| TCGA-CV-A463 | basal | YA |
| TCGA-CV-A464 | mesenchymal | YA |
| TCGA-CV-A465 | basal | YA |
| TCGA-CV-A468 | mesenchymal | YI |
| TCGA-CV-A6JD | mesenchymal | YA |
| TCGA-CV-A6JE | basal | YI |
| TCGA-CV-A6JN | basal | YI |
| TCGA-CV-A6JO | basal | YA |
| TCGA-CV-A6JT | basal | YA |
| TCGA-CV-A6JU | mesenchymal | YI |
| TCGA-CV-A6JY | basal | YI |
| TCGA-CV-A6JZ | mesenchymal | YI |
| TCGA-CV-A6K0 | mesenchymal | YI |
| TCGA-CV-A6K1 | basal | YI |
| TCGA-CV-A6K2 | basal | YA |
| TCGA-CX-7082 | classical | YI |
| TCGA-CX-7085 | mesenchymal | YI |
| TCGA-CX-7086 | atypical | YA |
| TCGA-CX-7219 | mesenchymal | YI |
| TCGA-CX-A4AQ | mesenchymal | YI |
| TCGA-D6-6515 | mesenchymal | YI |
| TCGA-D6-6516 | basal | YI |
| TCGA-D6-6517 | basal | YA |
| TCGA-D6-6823 | classical | YA |
| TCGA-D6-6824 | atypical | YI |
| TCGA-D6-6825 | basal | YI |
| TCGA-D6-6826 | mesenchymal | YA |
| TCGA-D6-6827 | atypical | YI |
| TCGA-D6-8568 | mesenchymal | YA |
| TCGA-D6-8569 | basal | YA |
| TCGA-D6-A4Z9 | basal | YI |
| TCGA-D6-A4ZB | classical | YI |
| TCGA-D6-A6EK | basal | YI |
| TCGA-D6-A6EM | mesenchymal | YI |
| TCGA-D6-A6EO | classical | YI |
| TCGA-D6-A6EP | mesenchymal | YI |
| TCGA-D6-A6EQ | basal | YI |
| TCGA-D6-A6ES | basal | YI |
| TCGA-D6-A74Q | atypical | YI |
| TCGA-DQ-5624 | basal | YA |
| TCGA-DQ-5625 | basal | YI |
| TCGA-DQ-5629 | classical | YI |
| TCGA-DQ-5630 | mesenchymal | YA |
| TCGA-DQ-7588 | basal | YA |
| TCGA-DQ-7589 | classical | YI |
| TCGA-DQ-7590 | atypical | YI |
| TCGA-DQ-7591 | atypical | YI |
| TCGA-DQ-7592 | basal | YA |
| TCGA-DQ-7593 | atypical | YI |
| TCGA-DQ-7594 | atypical | YI |
| TCGA-DQ-7595 | atypical | YI |
| TCGA-DQ-7596 | atypical | YI |
| TCGA-F7-7848 | atypical | YI |
| TCGA-F7-8298 | mesenchymal | YI |
| TCGA-F7-8489 | basal | YI |
| TCGA-F7-A50G | basal | YI |
| TCGA-F7-A50I | atypical | YI |
| TCGA-F7-A50J | basal | YA |
| TCGA-F7-A61S | basal | YA |
| TCGA-F7-A61V | atypical | YI |
| TCGA-F7-A61W | basal | YA |
| TCGA-F7-A620 | basal | YI |
| TCGA-F7-A622 | basal | YA |
| TCGA-F7-A623 | basal | YI |
| TCGA-F7-A624 | mesenchymal | YI |
| TCGA-H7-7774 | atypical | YI |
| TCGA-H7-8501 | basal | YA |
| TCGA-H7-8502 | basal | YI |
| TCGA-H7-A6C4 | basal | YA |
| TCGA-H7-A76A | mesenchymal | YI |
| TCGA-HD-7229 | basal | YA |
| TCGA-HD-7753 | basal | YA |
| TCGA-HD-7754 | atypical | YI |
| TCGA-HD-7831 | mesenchymal | YA |
| TCGA-HD-7832 | basal | YA |
| TCGA-HD-7917 | basal | YA |
| TCGA-HD-8224 | atypical | YI |
| TCGA-HD-8314 | atypical | YI |
| TCGA-HD-8634 | basal | YA |
| TCGA-HD-8635 | basal | YA |
| TCGA-HD-A4C1 | basal | YA |
| TCGA-HD-A633 | basal | YA |
| TCGA-HD-A634 | classical | YI |
| TCGA-HD-A6HZ | mesenchymal | YI |
| TCGA-HD-A6I0 | basal | YI |
| TCGA-IQ-7630 | basal | YA |
| TCGA-IQ-7631 | mesenchymal | YA |
| TCGA-IQ-7632 | basal | YA |
| TCGA-IQ-A61E | basal | YA |
| TCGA-IQ-A61G | classical | YI |
| TCGA-IQ-A61H | classical | YI |
| TCGA-IQ-A61I | atypical | YI |
| TCGA-IQ-A61J | basal | YA |
| TCGA-IQ-A61O | mesenchymal | YA |
| TCGA-IQ-A6SG | classical | YA |
| TCGA-IQ-A6SH | basal | YA |
| TCGA-KU-A66S | classical | YI |
| TCGA-KU-A66T | classical | YA |
| TCGA-KU-A6H7 | atypical | YI |
| TCGA-KU-A6H8 | atypical | YI |
| TCGA-MT-A51W | basal | YI |
| TCGA-MT-A51X | basal | YA |
| TCGA-MT-A67A | basal | YI |
| TCGA-MT-A67D | atypical | YI |
| TCGA-MT-A67F | basal | YI |
| TCGA-MT-A7BN | mesenchymal | YI |
| TCGA-MZ-A5BI | atypical | YI |
| TCGA-MZ-A6I9 | atypical | YI |
| TCGA-MZ-A7D7 | classical | YI |
| TCGA-P3-A5Q5 | atypical | YI |
| TCGA-P3-A5Q6 | mesenchymal | YA |
| TCGA-P3-A5QA | mesenchymal | YA |
| TCGA-P3-A5QE | atypical | YI |
| TCGA-P3-A5QF | atypical | YI |
| TCGA-P3-A6SW | atypical | YI |
| TCGA-P3-A6SX | basal | YI |
| TCGA-P3-A6T0 | classical | YA |
| TCGA-P3-A6T2 | basal | YA |
| TCGA-P3-A6T3 | basal | YA |
| TCGA-P3-A6T4 | classical | YI |
| TCGA-P3-A6T5 | basal | YA |
| TCGA-P3-A6T6 | atypical | YI |
| TCGA-P3-A6T7 | basal | YI |
| TCGA-P3-A6T8 | mesenchymal | YA |
| TCGA-QK-A64Z | basal | YA |
| TCGA-QK-A652 | basal | YA |
| TCGA-QK-A6IF | atypical | YI |
| TCGA-QK-A6IH | basal | YA |
| TCGA-QK-A6II | basal | YA |
| TCGA-QK-A6IJ | atypical | YA |
| TCGA-QK-A6V9 | atypical | YI |
| TCGA-QK-A6VB | classical | YI |
| TCGA-QK-A6VC | atypical | YI |
| TCGA-QK-A8Z7 | classical | YI |
| TCGA-QK-A8Z8 | classical | YI |
| TCGA-QK-A8Z9 | mesenchymal | YA |
| TCGA-QK-A8ZA | classical | YI |
| TCGA-QK-A8ZB | basal | YA |
| TCGA-QK-AA3J | atypical | YI |
| TCGA-QK-AA3K | mesenchymal | YI |
| TCGA-RS-A6TO | mesenchymal | YA |
| TCGA-T2-A6WZ | basal | YI |
| TCGA-T2-A6X0 | classical | YI |
| TCGA-T2-A6X2 | basal | YA |
| TCGA-T3-A92M | mesenchymal | YI |
| TCGA-TN-A7HI | atypical | YI |
| TCGA-TN-A7HJ | atypical | YI |
| TCGA-TN-A7HL | atypical | YI |
| TCGA-UF-A718 | basal | YA |
| TCGA-UF-A719 | classical | YI |
| TCGA-UF-A71A | classical | YI |
| TCGA-UF-A71B | classical | YI |
| TCGA-UF-A71D | atypical | YI |
| TCGA-UF-A71E | classical | YI |
| TCGA-UF-A7J9 | classical | YI |
| TCGA-UF-A7JA | classical | YI |
| TCGA-UF-A7JC | basal | YA |
| TCGA-UF-A7JD | mesenchymal | YA |
| TCGA-UF-A7JF | atypical | YI |
| TCGA-UF-A7JH | mesenchymal | YI |
| TCGA-UF-A7JJ | mesenchymal | YA |
| TCGA-UF-A7JK | basal | YA |
| TCGA-UF-A7JO | classical | YI |
| TCGA-UF-A7JS | basal | YI |
| TCGA-UF-A7JT | basal | YI |
| TCGA-UF-A7JV | mesenchymal | YA |
| TCGA-UP-A6WW | atypical | YI |
| TCGA-WA-A7GZ | classical | YA |
| TCGA-WA-A7H4 | mesenchymal | YA |
